# PD-1 blockade potentiates neoadjuvant chemotherapy in NSCLC via increasing CD127^+^ and KLRG1^+^ CD8 T cells

**DOI:** 10.1038/s41698-023-00384-x

**Published:** 2023-05-25

**Authors:** Zhenzhen Hui, Yulin Ren, Dong Zhang, Yulong Chen, Wenwen Yu, Jie Cao, Liang Liu, Tao Wang, Shanshan Xiao, Liuqing Zheng, Yue Pu, Feng Wei, Jian You, Xiubao Ren

**Affiliations:** 1 Tianjin Medical University Cancer Institute & Hospital, National Clinical Research Center for Cancer, Tianjin, 300060 China; 2 Tianjin’s Clinical Research Center for Cancer, Tianjin, 300060 China; 3 Key Laboratory of Cancer Immunology and Biotherapy, Tianjin, 300060 China; 4 Department of Biotherapy, Tianjin Medical University Cancer Institute & Hospital, Tianjin, 300060 China; 5 Department of Immunology, Tianjin Medical University Cancer Institute & Hospital, Tianjin, 300060 China; 6 Department of Lung Cancer, Tianjin Lung Cancer Center, Tianjin Medical University Cancer Institute & Hospital, Tianjin, 300060 China; 7 Wake Forest University, Winston‐Salem, NC USA; 8 Department of R&D, Hangzhou Repugene Technology Co., Ltd., Hangzhou, 311100 China

**Keywords:** Non-small-cell lung cancer, Cancer immunotherapy, Cancer microenvironment

## Abstract

The combination of PD-1 blockade with neoadjuvant chemotherapy (NAC) has achieved unprecedented clinical success in non-small cell lung cancer (NSCLC) compared to NAC alone, but the underlying mechanisms by which PD-1 blockade augments the effects of chemotherapy remain incompletely elucidated. Single-cell RNA sequencing was performed on CD45^+^ immune cells isolated from surgically resected fresh tumors of seven NSCLC patients receiving NAC or neoadjuvant pembrolizumab and chemotherapy (NAPC). Multiplex fluorescent immunohistochemistry was performed on FFPE tissues before and after NAC or NAPC from 65 resectable NSCLC patients, and results were validated with GEO dataset. NAC resulted in an increase only of CD20^+^ B cells, whereas NAPC increased the infiltration of CD20^+^ B cells, CD4^+^ T cells, CD4^+^CD127^+^ T cells, CD8^+^ T cells, CD8^+^CD127^+^ and CD8^+^KLRG1^+^ T cells. Synergistic increase in B and T cells promotes favorable therapeutic response after NAPC. Spatial distribution analysis discovered that CD8^+^ T cells and their CD127^+^ and KLRG1^+^ subsets were in closer proximity to CD4^+^ T/CD20^+^ B cells in NAPC versus NAC. GEO dataset validated that B-cell, CD4, memory, and effector CD8 signatures correlated with therapeutic responses and clinical outcomes. The addition of PD-1 blockade to NAC promoted anti-tumor immunity through T and B cells recruitment in the tumor microenvironment and induced tumor-infiltrating CD8^+^ T cells skewed toward CD127^+^ and KLRG1^+^ phenotypes, which may be assisted by CD4^+^ T cells and B cells. Our comprehensive study identified key immune cell subsets exerting anti-tumor responses during PD-1 blockade therapy and that may be therapeutically targeted to improve upon existing immunotherapies for NSCLC.

## Introduction

Lung cancer is one of the most common malignancies and the leading cause of cancer-related death worldwide^1^, of which non-small cell lung cancer (NSCLC) account for about 80–85%^2^. Patients with resectable NSCLC are routinely treated with surgical resection, however, 30–60% experience postsurgical relapse and ultimately die of their disease^3^. Neoadjuvant therapy is a promising therapeutic option to shrink tumor mass, increase surgical opportunity, and eliminate invisible micrometastases early. Neoadjuvant chemotherapy (NAC) followed by surgery significantly improves the 5-year survival rate of NSCLC patients by 5%, from 40% to 45%, compared to surgery alone^4^.

Immunotherapy, represented by immune checkpoint blockades (ICBs), has revolutionized the treatment strategies of multiple cancer types, including NSCLC. Recent clinical trials are exploring the use of ICBs alone or in combination with chemotherapy as a neoadjuvant treatment regimen in patients with NSCLC, inspired by their remarkable clinical efficacy for the treatment of advanced NSCLC^5–7^. Two single-arm clinical studies support the addition of ICBs to chemotherapy in this subset of patients, with high major pathological response (MPR) rates and manageable treatment-related toxicity^5,6^. Checkmate 816 study (NCT02998528), a phase 3 trial to evaluate the efficacy and safety of neoadjuvant chemotherapy plus nivolumab versus chemotherapy in patients with resectable NSCLC, revealed nivolumab plus chemotherapy dramatically outperforms standard platinum-based chemotherapy with respect to event-free survival (30.2 months versus 20.8 months) and pathological complete response (pCR; 24.0% versus 2.2%) and had no adverse effect on surgical feasibility or surgical outcomes^8^. NADIM II (NCT03838159), an open-label, randomized, two-arm, phase II, multi-center clinical trial, confirmed neoadjuvant nivolumab plus chemotherapy significantly increased the pCR (36.2% vs 6.8%) and MPR rates (52.0% vs 14.0%) compared to chemotherapy, as well as the feasibility of surgery, with a moderate increase in grade 3-4 toxicity^9^.

As mentioned above, the combination of ICBs and chemotherapy is superior to chemotherapy alone. Researchers have depicted ICBs-induced changes in the immune microenvironment at gene level using single-cell sequencing of pre- and post-treatment biopsies from patients receiving anti-PD1 or anti-PD-L1 therapy in multiple tumor types, including melanoma, basal or squamous cell carcinoma, and breast cancer^10–13^. However, the underlying mechanisms by which PD-1 blockade augments the therapeutic effect of chemotherapy remain incompletely understood. Here, we performed a comprehensive analysis of tumor-infiltrating immune cells before and after NAC or neoadjuvant pembrolizumab and chemotherapy (NAPC) for resectable NSCLC by single-cell RNA sequencing (scRNA-seq) and multiplex fluorescent immunohistochemistry (mIHC) and evaluated their relationship with therapeutic response and clinical outcomes. Our study revealed the distinct impacts of NAC or NAPC on tumor immune microenvironment and identified key immune cell subsets by which PD-1 blockade enhances the antitumor immune response to chemotherapy, providing new insights for improving the efficiency of immunotherapy and chemotherapy in NSCLC.

## Results

### NAPC improves pathological response and survival in NSCLC patients

A total of 30 patients in the NAC group and 35 patients in the NAPC group were included in our mIHC study. The clinicopathologic characteristics of the included patients are described in Table 1. Characteristics of patients were generally well balanced between the two groups except for type of resection (*P* = 0.078), with a slightly higher percentage of patients in the NAC group undergoing pneumonectomy. This may be due to the inability of chemotherapy to shrink the tumor to a size suitable for lobectomy. Squamous carcinoma was the predominant pathological type in both NAC and NAPC groups, with more than 80% being male and approximately 20% being never smokers. Patients treated with NAPC obtained a higher MPR than NAC, 65.7% (23 of 35 patients) and 20% (6 of 30 patients), respectively (Table 1, *P* < 0.0001; Supplementary Fig. 1a). Moreover, 16 patients (45.7%) achieved pCR in the NAPC group while no patients exhibited pCR in the NAC group (Supplementary Fig. 1a). Patients receiving NAPC had a better prognosis, as evidenced by the significantly longer DFS (Hazard ratio=0.3216, 95% CI 0.1455–0.7105, *P* = 0.0031; Supplementary Fig. 1b) and OS (Hazard ratio=0.2368, 95% CI 0.1043–0.5376, *P* = 0.0016; Supplementary Fig. 1c) in the NAPC group than that in the NAC group.

**Table 1:** Clinocopathological characteristics of NSCLC patients treated with NAC or NAPC.

Characteristics	NAC (*N* = 30)	NAPC (*N* = 35)	*p* value
Age (years)			0.612
≤60	13 (43.3%)	13 (37.1%)	
>60	17 (56.7%)	22 (62.9%)	
Sex			0.791
Female	5 (16.7%)	5 (14.3%)	
Male	25 (83.3%)	30 (85.7%)	
Smoking status			0.314
Never	6 (20.0%)	6 (17.1%)	
Former	1 (3.3%)	5 (14.3%)	
Current	23 (76.7%)	24 (68.6%)	
Tumor status			0.794
T1	3 (10.0%)	3 (8.6%)	
T2	16 (53.4%)	21 (60.0%)	
T3	7 (23.3%)	5 (14.3%)	
T4	4 (13.3%)	6 (17.1%)	
Nodal status			0.666
N0	6 (20.0%)	11 (31.4%)	
N1	4 (13.3%)	3 (8.6%)	
N2	19 (63.4%)	19 (54.3%)	
N3	1 (3.3%)	2 (5.7%)	
cTNM			0.929
II	7 (23.3%)	7 (20.0%)	
IIIA	21 (70.0%)	26 (74.3%)	
IIIB	2 (6.7%)	2 (5.7%)	
Pathology			0.631
Squamous	17 (56.7%)	23 (65.7%)	
Adenocarcinoma	11 (36.7%)	9 (25.7%)	
Large-cell	2 (6.6%)	2 (5.7%)	
Sarcomatoid	0 (0%)	1 (2.9%)	
Neoadjuvant regimen			0.471
PTX + CBP	18 (60.0%)	–	
PEM + CBP	12 (40.0%)	–	
PTX + CBP+Pembro	–	24 (68.6%)	
PEM + CBP+Pembro	–	11 (31.4%)	
Type of resection			0.078
Pneumonectomy	7 (23.3%)	3 (8.6%)	
Lobectomy	20 (66.7%)	22 (62.9%)	
Sleeve lobectomy	3 (10%)	10 (28.5%)	
Pathological response			<0.0001
non-MPR	24 (80.0%)	12 (34.3%)	
MPR	6 (20.0%)	23 (65.7%)	
Adjuvant therapy			0.959
Yes	25 (83.3%)	29 (82.9%)	
No	5 (16.7%)	6 (17.1%)	

*NSCLC* non-small cell lung cancer, *NAC* neoadjuvant chemotherapy, *NAPC* neoadjuvant pembrolizumab and chemotherapy, *TNM* Tumor Node Metastasis, *PTX* paclitaxel, *CBP* carboplatin, *PEM* pemetrexed, *MPR* major pathological response.

### Single-cell immune profiling of NSCLC patients treated with NAC or NAPC

**Fig. 1:**
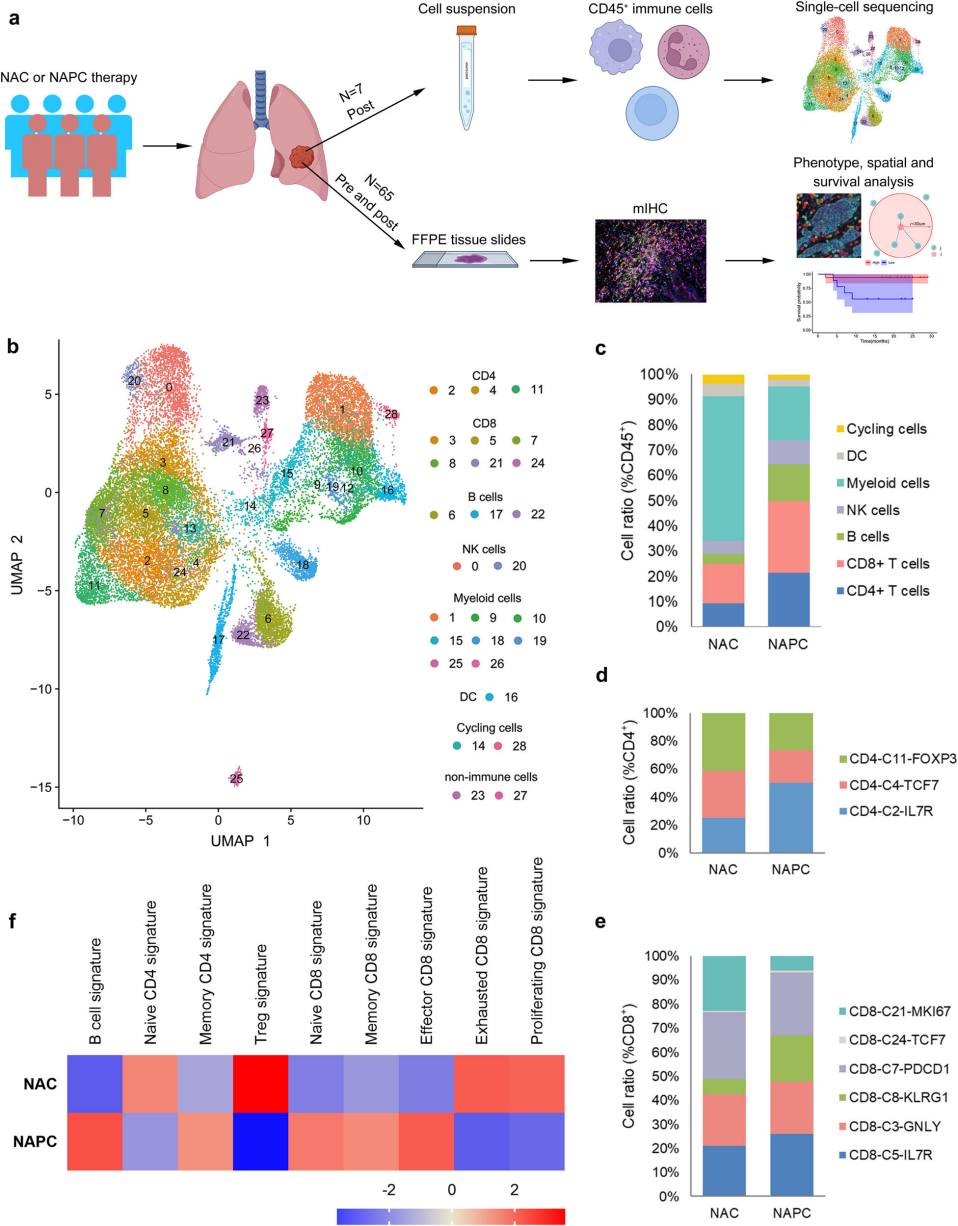
Single-cell map of immune cells from NSCLC patients treated with neoadjuvant NAC and NAPC therapy. **a** Schematic overview of the study design (Created with BioRender.com). CD45^+^ immune cells isolated from surgical resected fresh tumors of seven patients receiving two cycles NAC or NAPC were subjected to single-cell sequencing. Paired FFPE tissues at biopsy before neoadjuvant therapy and at surgery after neoadjuvant therapy were collected for mIHC staining. **b** UMAP display of immune cells in NSCLC patients treated with neoadjuvant NAC and NAPC therapy. Each dot corresponds to one single cell, colored according to cell cluster. **c** Immune cell composition in neoadjuvant NAC and NAPC therapy. **d**–**e** Comparison of CD4^+^ T cell and CD8^+^ T cell clusters proportions between NAC and NAPC groups. **f** GSEA analysis of different lymphocyte subpopulation signatures in NAC and NAPC.

To investigate alterations in tumor-infiltrating immune cells after the addition of PD-1 blockade to NAC, we carried out scRNA-seq on resected tumors from six patients receiving NAPC and one patient receiving NAC who were diagnosed with resectable NSCLC (Fig. 1a, Supplementary Table 1). After stringent quality filtering, we obtained 26,861 single-cell transcriptomes, with 4,057 for NAC and 22,804 for NAPC. Unsupervised clustering of the single-cell transcriptomes generated 29 distinct clusters displayed by uniform manifold approximation and projection (UMAP), including three CD4^+^ T cells clusters, six CD8^+^ T cells clusters, three B cells clusters, two NK cells clusters, nine myeloid cells clusters, two proliferating cell cluster, and two less-abundant non-immune cells clusters consisted of endothelial cells and epithelial cells (Fig. 1b). These two non-immune cells clusters were excluded from the subsequent analysis due to their low content and were not the focus of our attention. We calculated the percentage of each immune cell cluster out of all immune clusters and found that patients with the two treatment regimens displayed distinct immune cell composition, with myeloid cells being the predominant cells in patients receiving NAC, whereas CD4^+^ T cells, CD8^+^ T cells, and B cells were more abundant in patients treated with NAPC (Fig. 1c).

Subsequent analysis of T cell clusters revealed that CD8-C3-GNLY and CD8-C8-KLRG1 clusters characterized by expression of killer cell lectin-like receptor G1 (KLRG1) along with high expression of cytotoxic and effector signature genes such as GZMK, GZMH, GNLY, NKG7, GZMB, CTSW, CST7, and PRF1, resembling effector CD8^+^ T cells, while CD8-C5-IL7R cluster was enriched for the memory marker IL7R (CD127). The CD8-C7-PDCD1 has hallmarks of exhausted cells and central memory cells, including expression of the checkpoint protein PDCD-1 and the transcription factor TCF7, with stem-cell-like properties^14^ and was defined as progenitor-like precursor exhausted cells. CD8-C21-MKI67 exhibited proliferative properties, and CD8-C24-TCF7 was defined as a naive phenotype (Supplementary Fig. 2, Supplementary Fig. 3). Comparison of cluster composition between NAC and NAPC identified that CD4-C2-IL7R, CD8-C5-IL7R and CD8-C8-KLRG1 clusters tended to increase in NAPC tumor lesions after neoadjuvant therapy, while CD4-C11-FOXP3 clusters tended to decrease (Fig. 1d, e). This trend in cell ratios reflects the possibility that CD127-expressing T cells and KLRG1-expressing CD8^+^ T cells are the main immune cell types mediating anti-tumor immunity after the addition of PD-1 blockade to neoadjuvant chemotherapy. We compared differential expression genes (DEGs) in CD8^+^KLRG1^+^ and CD8^+^KLRG1^-^ T cells and found that cytotoxic genes such as CST7, CTSW, GZMA, GZMH, NKG7, and PRF1 were highly expressed in CD8^+^KLRG1^+^ T cells. Go functional enrichment analysis showed that CD8^+^KLRG1^+^ T cells were mainly concentrated on cellular defense response, response to hydrogen peroxide, cytolysis, and cell chemotaxis, whereas CD8^+^KLRG1^-^ T cells were mainly enriched in transmembrane receptor protein serine/threonine kinase signaling pathways, cellular iron ion homeostasis, NADH regeneration, and canonical glycolysis (Supplementary Fig. 4).

To in-depth explore the transcriptome characteristics between NAC and NAPC, We applied gene set enrichment analysis (GSEA) to detect signatures of each lymphocyte subpopulations (Supplementary Table 2) and demonstrated that B-cell, memory CD4, naïve CD8, memory CD8 and effector CD8 signatures were much abundant in NAPC tumor lesions, whereas Treg and exhausted CD8 signatures were more enriched in the NAC group (Fig. 1f), indicating a potentially important role for these immune cell types in mediating anti-tumor immune responses after neoadjuvant therapy.

### Transcriptome characterization of CD8^+^ T cells after neoadjuvant therapy

**Fig. 2:**
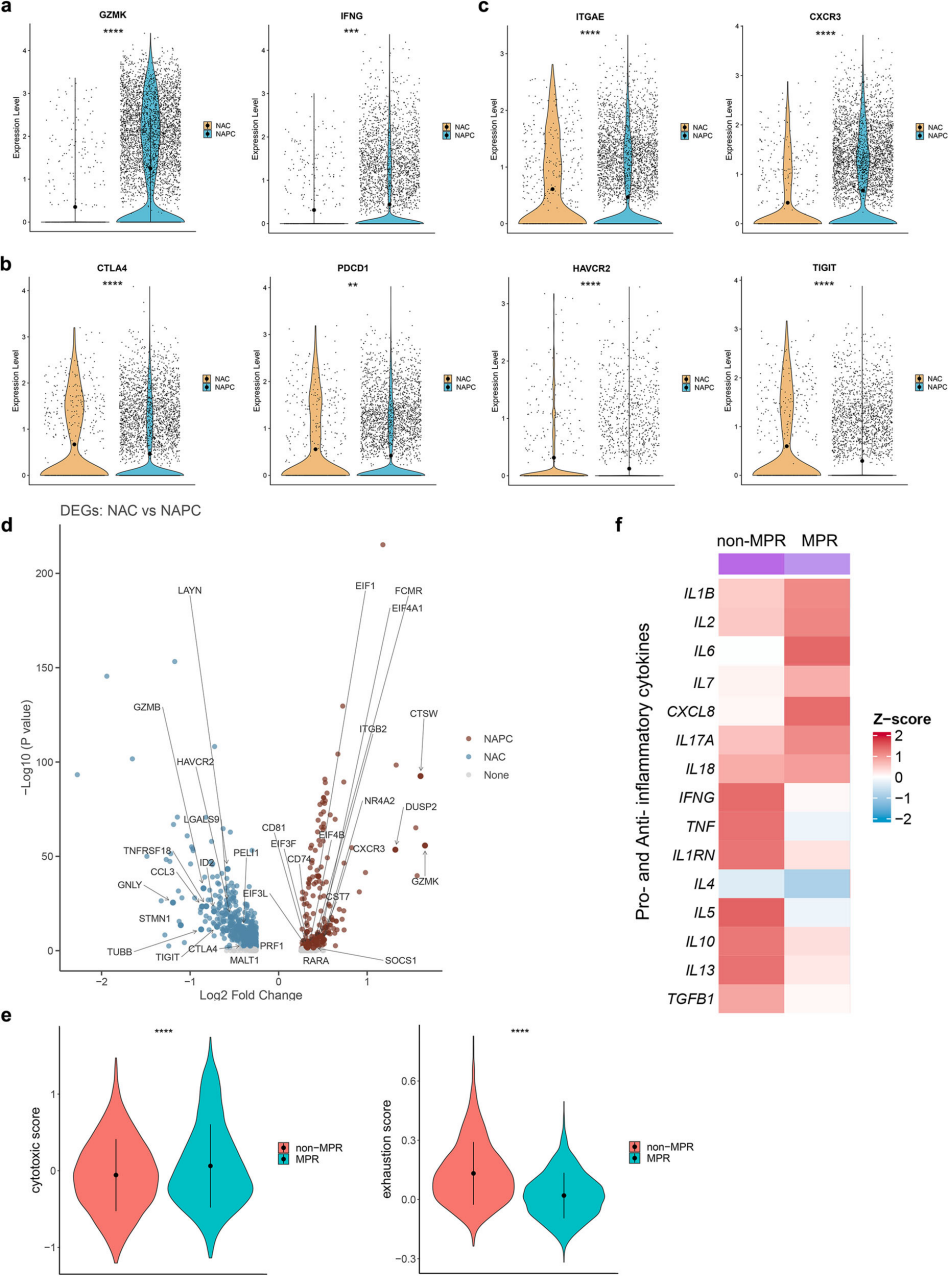
Transcriptome profiling of CD8^+^ T-cell clusters after neoadjuvant therapy. **a**–**c** Violin plot showing functional gene expression levels of tumor-infiltrating CD8^+^ T cells in NAC and NAPC. Data are presented as mean. **d** Volcano plot showing comparison of DEGs of CD8^+^ T cells between NAC and NAPC. Inclusion criteria: Log2FC > 0.25, *p* < 0.05, min.pct > 0.1. **e** Comparison of CD8 cytotoxic score and exhausted score between non-MPR and MPR in NAPC estimated by the “AddModuleScore” function in Seurat. Data are presented as mean ± SD. **f** Heatmap displayed the expression levels of pro- and anti-inflammatory cytokines in tumor tissues of non-MPR and MPR patients.

The functional status of tumor-infiltrating CD8^+^ T cells was also different between NAC and NAPC after neoadjuvant therapy. We compared the functional gene expression levels of CD8^+^ T cells from NAC and NAPC, including co-stimulation molecules (ICOS, CD28, TNFRSF9), effector molecules (GZMK, GZMB, PRF1, IFNG, TNF), exhausted markers (TOX, PDCD1, CTLA4, TIGIT, HAVCR2, LAG3, TIM3), tissue-resident markers (ITGAE, CD69, ENTPD1), as well as chemokine receptors (CCR5, CXCR3, CXCR4, CXCR6, CX3CR1)^15^. We found that CD8^+^ T cells in the NAPC group highly expressed cytotoxic genes GZMK and IFNG and lowly expressed exhausted genes such as CTLA4, PDCD1, HAVCR2, and TIGIT compared with NAC (Fig. 2a, b). We also noted that the tissue-resident marker ITGAE (CD103) was highly expressed in CD8^+^ T cells in the NAC group, indicating the presence of more tissue-resident CD8^+^ T cells in NAC tumor lesions. In contrast, CD8^+^ T cells in the NAPC group highly expressed the chemokine receptor CXCR3 (Fig. 2c), which is a receptor for chemokines CXCL9, CXCL10, and CXCL11 and is responsible for recruiting CD8^+^ T cells to infiltrate into tumor tissues, proposing the potential that the combination of PD-1 blockade to NAC may attract more CD8^+^ T cells into tumor tissue to exert anti-tumor effects^16^. DEGs analysis of CD8^+^ T cells between NAC and NAPC also confirmed that CD8^+^ T cells in the NAC group highly expressed exhausted genes such as LAYN, ID2, HAVCR2, and TIGIT, whereas CD8^+^ T cells in the NAPC group highly expressed effector genes such as CTSW, GZMK, DUSP2, CST7, and eukaryotic translation initiation factors EIF1, EIF3, EIF4A1, and EIF4B (Fig. 2d).

By examining signature genes defined previously^17^, we compared the functional status of CD8^+^ T cells in non-MPR and MPR patients in the NAPC group using the “AddModuleScore” in Seurat^18^ and found that CD8^+^ T cells in patients who obtained MPR after treatment had the higher cytotoxic score and lower exhausted score (Fig. 2e; Supplementary Table 3). The expression level of intra-tumor inflammatory cytokines^19^ was also associated with the therapy response in NAPC, with anti-inflammatory cytokines (such as IL1RN, IL5, IL10, IL13 and TGFB1) enriched in non-MPR and pro-inflammatory cytokines (such as IL1B, IL2, IL6, IL7, IL8, IL17A, and IL18) enriched in MPR tumor lesions (Fig. 2f).

### Increased tumor-infiltrating CD127^+^ and KLRG1^+^ CD8 T cells after NAPC

To further validate the results of scRNA-seq, we performed mIHC on Formalin-fixed and paraffin-embedded (FFPE) tumor tissues from 65 resectable NSCLC patients before and after neoadjuvant therapy with either NAC (16 paired pre-NAC and post-NAC and 14 unpaired post-NAC samples) or NAPC (18 paired pre-NAPC and post-NAPC, 5 unpaired pre-NAPC and 12 unpaired post-NAPC samples). We mainly focus on T cells with different differentiation states and functional properties as well as B cells based on our scRNA-seq results. We found no difference in the proportions of CD20^+^ B cells, CD4^+^ T cells, CD4^+^CD127^+^ T cells, CD4^+^FoxP3^+^ Tregs, CD8^+^ T cells, CD8^+^CD127^+^ T cells, and CD8^+^KLRG1^+^ T cells infiltration between pre-NAC and pre-NAPC (Supplementary Fig. 5a, b), confirming that the altered cell proportions after neoadjuvant therapy were not due to the discrepancy in the pre-treatment samples at baseline. Moreover, the expression level of PD-L1 in pre-treatment tumor was not associated with the level of intra-tumoral lymphocyte infiltration in NAPC, and did not correlate with therapeutic response and tumor necrosis rate (Supplementary Table 4, Supplementary Fig. 5c, d).

**Fig. 3:**
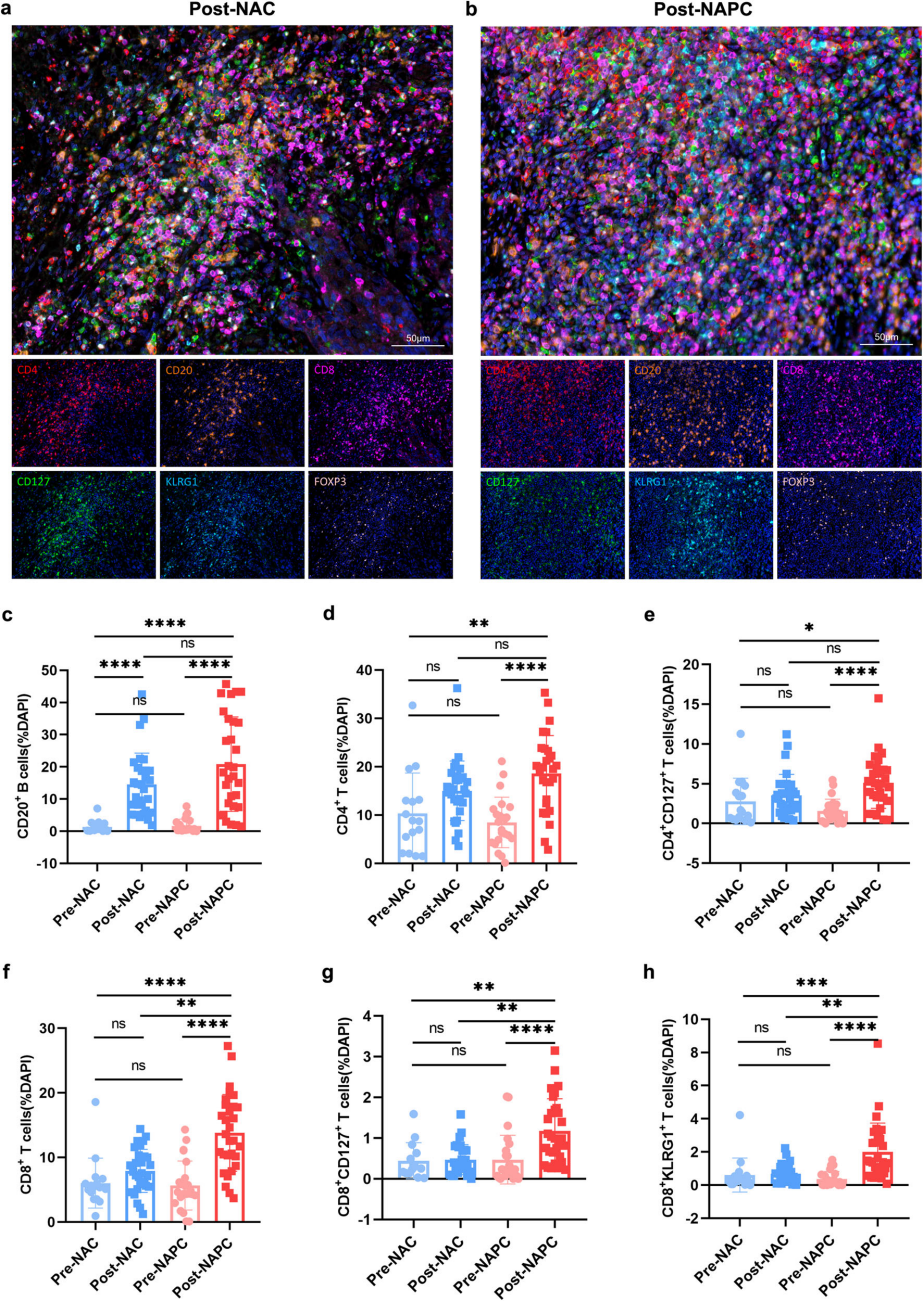
CD8^+^ T cells and their CD127^+^ and KLRG1^+^ subsets were enriched in NAPC. **a**, **b** Representative mIHC staining image of post-treatment samples in the NAC and NAPC groups (Image taken under 20X). **c**–**h** Comparison of CD20^+^ B cells, CD4^+^ T cells, CD4^+^CD127^+^ T cells, CD8^+^ T cells, CD8^+^CD127^+^ T cells, and CD8^+^KLRG1^+^ T cells in pre- and post-treatment samples in the NAC and NAPC groups. Pre-NAC: *n* = 16, Post-NAC: *n* = 30, Pre-NAPC: *n* = 23, Post-NAPC: *n* = 30. Data are presented as mean ± SD. *P* values were determined by Kruskal-Wallis test, * *P* < 0.05; ** *P* < 0.01; *** *P* < 0.001; **** *P* < 0.0001 ns not significant.

We investigated the tumor-infiltrating lymphocytes (TILs) changes before and after treatment in the NAC and NAPC groups separately. We demonstrated that except for CD20^+^ B cells, the ratios of CD4^+^ T cells, CD4^+^CD127^+^ T cells, CD8^+^ T cells, CD8^+^CD127^+^ T cells, and CD8^+^KLRG1^+^ T cells did not change significantly in post-NAC versus pre-NAC, reflecting the limited immune activation capacity of chemotherapy alone. In contrast, post-NAPC exhibited a synergistic increase in the proportion of CD20^+^ B cells, CD4^+^ T cells, CD4^+^CD127^+^ T cells, CD8^+^ T cells, CD8^+^CD127^+^ T cells and CD8^+^KLRG1^+^ T cells compared with pre-NAPC (Fig. 3a–h). Pairwise analysis of self-matched pre- and post-treatment samples from the NAC and NAPC groups also confirmed these findings, indicating the extensive activation and enrichment of lymphocytes after adding PD-1 blockade to neoadjuvant chemotherapy (Supplementary Fig. 6). Furthermore, CD8^+^ T cells, CD8^+^CD127^+^ T cells, and CD8^+^KLRG1^+^ T cells ratios were significantly higher in post-NAPC compared to post-NAC (Fig. 3f–h), indicating that CD8^+^ T cells and their CD127^+^ and KLRG1^+^ subsets were preferentially enriched after adding PD-1 blockade to NAC.

We also explored the relationship between tumor size and the level of TILs and demonstrated that tumor size was negatively correlated with CD4^+^ T cells, CD4^+^CD127^+^ T cells and Treg cells infiltration in NAC (Supplementary Table 5, Supplementary Fig. 5e), while tumor size was not correlated with lymphocyte infiltration in NAPC. This may be because PD-1 blockade therapy promoted more infiltration of TILs into tumor tissues, and this effect of PD-1 blockade overwhelmed the influence of tumor size on TILs infiltration.

### Synergistic increase in B and T cells promotes favorable therapeutic response after NAPC

**Fig. 4:**
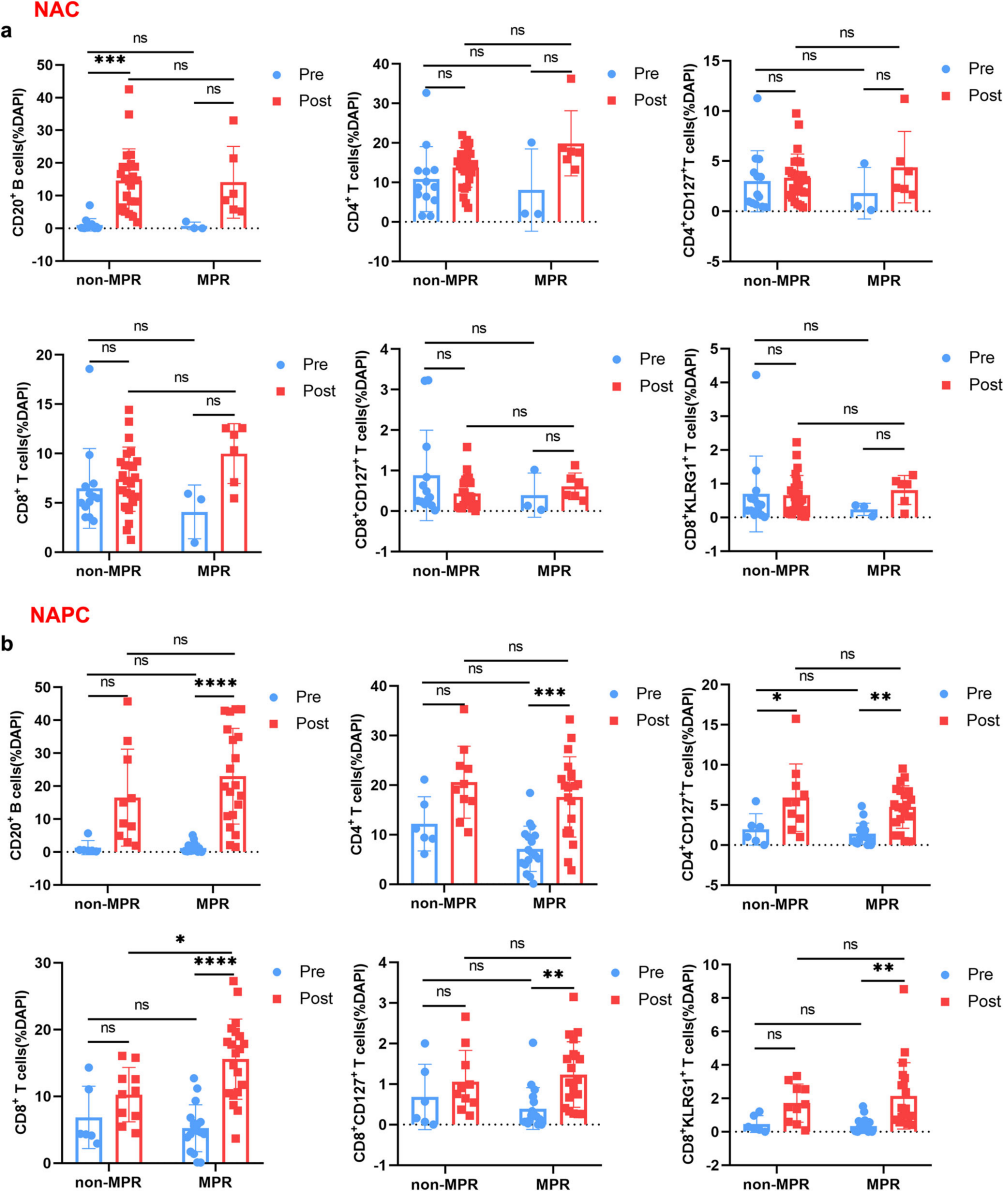
Comparison of TILs in non-MPR and MPR patients before and after treatment in NAC and NAPC groups. **a** Comparison of TILs in non-MPR and MPR patients pre- and post-treatment in NAC. **b** Comparison of TILs in non-MPR and MPR patients pre- and post-treatment in NAPC. NAC: Pre-NR *n* = 13, Post-NR *n* = 24, Pre-R *n* = 3, Post-R *n* = 6; NAPC: Pre-NR *n* = 6, Post-NR *n* = 10, Pre-R *n* = 17, Post-R *n* = 20. Data are presented as mean ± SD. *P* values were determined by two-way ANOVA with multiple comparisons, * *P* < 0.05; ** *P* < 0.01; *** *P* < 0.001; **** *P* < 0.0001; ns not significant. NR, non-MPR; R, MPR.

We explored the cell ratio changes in non-MPR and MPR patients before and after treatment in both NAC and NAPC group. We found that in the NAC group, dynamics of cell ratios before and after treatment were not associated with therapeutic response in either non-MPR or MPR patients, including elevated CD20 ratio after NAC. Whereas in the NAPC group, compared to pre-NAPC, post-NAPC CD20^+^ B cells, CD4^+^ T cells, CD8^+^ T cells, CD8^+^CD127^+^ T cells, and CD8^+^KLRG1^+^ T cells were significantly elevated in MPR, but not in non-MPR patients (Fig. 4a, b), suggesting that the co-increase of these subpopulations could coordinate and interplay with each other to promote anti-tumor immune responses.

### Influence of different chemotherapy regimens on TILs

Different chemotherapeutic agents may affect the level and immune phenotype of TILs after therapy. We next investigated TILs before and after different chemotherapy regimens and showed that the alterations in TILs were mostly independent of treatment regimen and pathological type, except for CD4^+^CD127^+^ T cells, which were slightly higher in pemetrexed-treated patients (adenocarcinoma) than in paclitaxel-treated patients (squamous cell carcinoma) in the NAC group (*P* = 0.0116; Supplementary Table 6–7). However, this difference disappeared when concomitantly combined with pembrolizumab, probably because PD-1 blockade was able to affect the infiltration level of CD4^+^CD127^+^ T cells to a greater extent, thus compensating for the relatively small difference caused by chemotherapeutic regimens.

### Spatial distribution of CD127^+^/KLRG1^+^ CD8 T cells and CD4^+^ T/CD20^+^ B cells

**Fig. 5:**
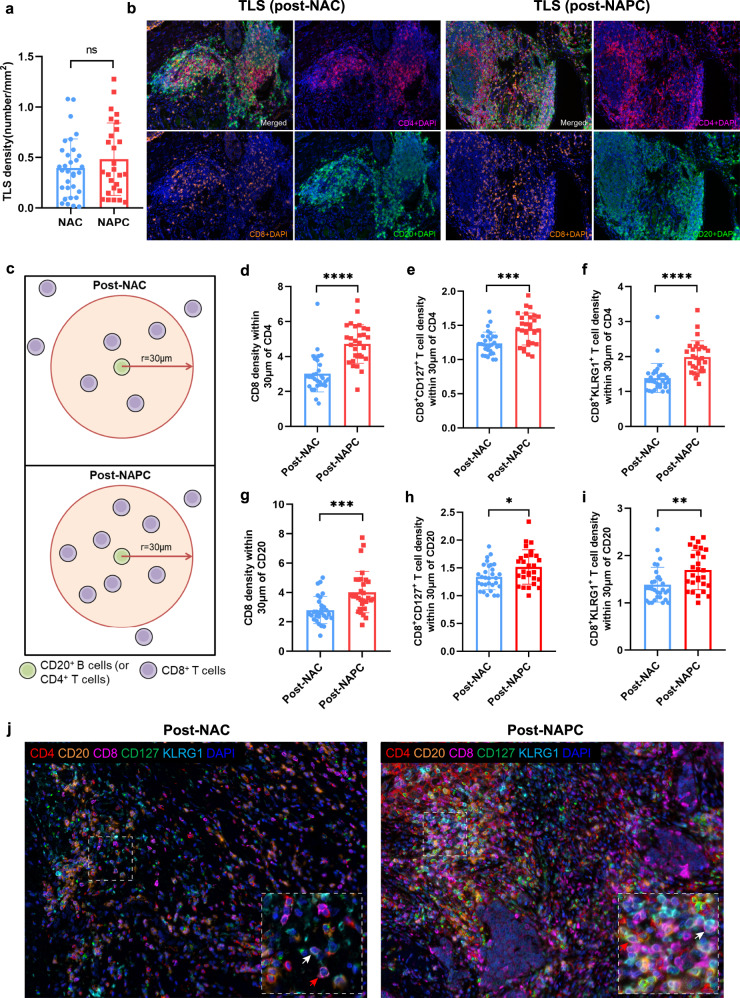
Spatial distribution patterns of CD8^+^ T cells and CD4^+^ T/CD20^+^ B cells. **a** The TLS density was slightly higher in post-NAPC than post-NAC. **b** Representative TLSs in post-treatment samples of NAC and NAPC. The TLSs in post-NAC were smaller and mostly located in the periphery of the tumor which presented like lymphoid aggregates, while the TLSs in post-NAPC were mature, organized TLSs with defined T cell/B cell zones. **c** Depiction of methodology for spatial analyses performed. Densities of interest cell being within a certain radius to a reference cell were calculated. **d**–**f** More CD8^+^ T cells, CD8^+^CD127^+^ T cells and CD8^+^ KLRG1^+^ T cells are present within 30um of CD4^+^ T cells in NAPC patients. **g**–**i** More CD8^+^ T cells, CD8^+^CD127^+^ T cells and CD8^+^ KLRG1^+^ T cells are present within 30um of CD20^+^ B cells in NAPC patients. **j** Representative NAC and NAPC images showing the distance distribution of CD4^+^ T cells, CD20^+^ B cells and CD8^+^ T cells, white arrows point to CD8^+^KLRG1^+^ T cells, red arrows point to CD8^+^CD127^+^ T cells (Image taken under 20X). Post-NAC: *n* = 30, Post-NAPC: *n* = 30. Data are presented as mean ± SD. *P* values were determined by Mann-Whitney test, *P < 0.05; **P < 0.01; ***P < 0.001; ****P < 0.0001.

Tumor-infiltrating immune cells interacted with each other to maintain their phenotype and function^20^, especially in the tertiary lymphoid structures (TLSs)^21^. The density of TLSs showed the higher trend in post-NAPC than in post-NAC, although it did not reach statistical significance due to the higher necrosis rate of pCR patients in post-NAPC (Fig. 5a, b; *P* = 0.3064). We noticed that the presence of CD127^+^/KLRG1^+^ CD8 T cells was positively correlated with that of CD20^+^ B cells and CD4^+^ T cells in both post-NAC and post-NAPC tumor tissues (Supplementary Fig. 7), indicating a possible intercellular interaction between these cell types. To quantify the interaction between these cells, we used bivariate K(r) function^22^ to characterize the spatial distributions of each two phenotype of TILs in our mIHC panel. Since the radius *r* = 30 μm is considered ideal for calculating the spatial relationship between two cell populations (Fig. 5c)^22,23^. We confirmed a significant increase in CD8^+^ T cells, CD8^+^CD127^+^ T cells, and CD8^+^KLRG1^+^ T cells within 30 μm around CD20^+^ B cells and CD4^+^ T cells in the post-NAPC group compared with post-NAC (Fig. 5d–i), which represents an increased probability of cell-cell contact in the post-NAPC tissues. Representative images were displayed in Fig. 5j. The spatial distribution of CD8^+^ T and CD8^+^CD127^+^ T cells with CD4^+^ T/CD20^+^ B cells showed an elevated trend in MPR patients in both NAC and NAPC, whereas CD8^+^KLRG1^+^ T cells exhibited an elevated trend in non-MPR patients in the NAPC group (Supplementary Fig. 8a–f), which requires a larger cohort to verify. There was no difference in the spatial distribution between CD4^+^ T cells, CD4^+^CD127^+^ T and CD20^+^ B cells (Supplementary Fig. 8g–i). The spatial distribution patterns that CD8^+^ T cells, CD127^+^/KLRG1^+^ CD8 T cells within the vicinity of CD20^+^ B and CD4^+^ T cells were consistent with the fact that CD8^+^ T cells differentiation and function requires the assistance of CD4^+^ T cells and B cells^20,24^.

### Potential ligand-receptor interactions between CD127^+^/KLRG1^+^ CD8 T cells and CD4^+^ T/CD20^+^ B cells

To further assess the specific ligand-receptor pairs of CD4^+^ T or B cells interacting with CD8^+^ T cells, we used CellChat^25^ to quantitatively characterize the predicted intercellular communication networks in our scRNA profiles. We predicted that CD4^+^ T cells may interact with CD8^+^CD127^+^ T and CD8^+^KLRG1^+^ T cells via the CXCL12-CXCR4 and IL7-IL7R pathways, and B cells may interact with CD8^+^CD127^+^ T and CD8^+^KLRG1^+^ T cells via BTLA-TNFRSF14 pathway (Supplementary Fig. 9). These findings implicated that CD4^+^ T and B cells may recruit CD8 to neighborhood via the CXCL12-CXCR4 signaling pathway^26,27^ and regulate CD8^+^ T cell function by secreting cytokines such as IL-7 (essential for T-cell development and survival) or through receptor-ligand interactions. Although CellChat analysis does not definitely demonstrate that the cells are interacting, it provides clues to possible intercellular interactions and potential receptor-ligand interaction, which need to be verified by further functional experiments.

### Prognostic impact of pre- and post-treatment TILs on patient survival

**Fig. 6:**
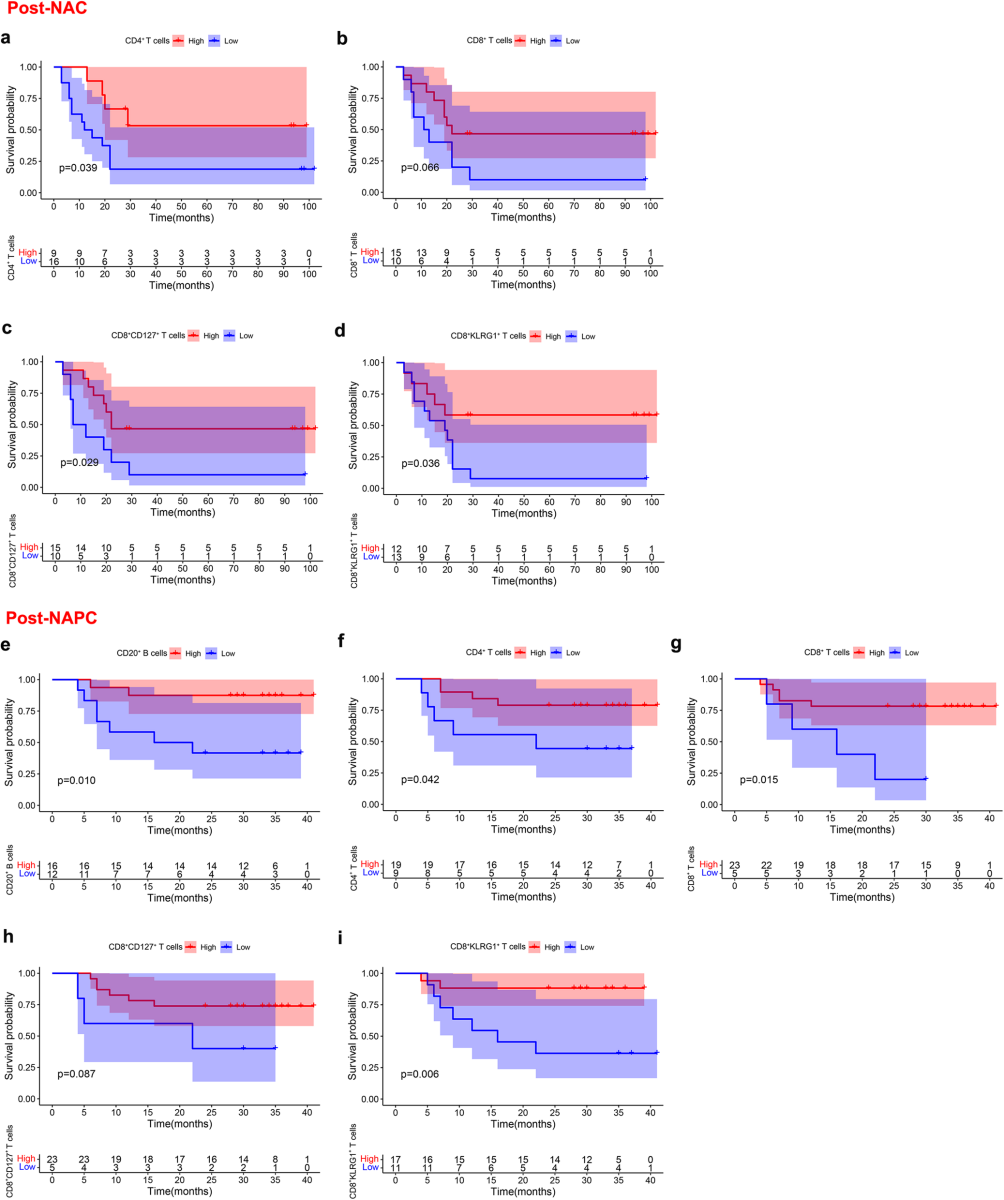
Correlation of post-treatment TILs with clinical outcome. **a**–**d** Kaplan–Meier estimates of DFS for NAC patients according to the post-treatment level of CD4^+^ T cells, CD8^+^ T cells, CD8^+^CD127^+^ T cells and CD8^+^KLRG1^+^ T cells. **e**–**i** Kaplan–Meier estimates of DFS for NAPC patients according to the post-treatment level of CD20^+^ B cells, CD4^+^ T cells, CD8^+^ T cells, CD8^+^CD127^+^ T cells and CD8^+^KLRG1^+^ T cells. Patients were divided into high and low groups using “survminer” package of the R software.

The TILs played a major role for the clinical outcome of patients^28^. To better identify the contribution of each lymphocyte population to clinical outcomes in the pre-and post-treatment tumor samples, we analyzed their prognostic impact on patient survival. We demonstrated that higher pre-treatment CD4^+^FoxP3^+^ T cells predicted shorter DFS in NAC (Supplementary Fig. 10a), whereas higher pre-treatment CD20^+^ B cells, CD8^+^ T cells, and CD8^+^CD127^+^ T cells predicted longer DFS in NAPC (Supplementary Fig. 10b–d), suggesting that pre-treatment B cells, CD8^+^ T cells, and CD8^+^CD127^+^ T cells are the mainstay in mediating the anti-tumor immune responses during PD-1 blockade therapy^20,29,30^. For post-treatment samples, higher CD4^+^ T cells, CD8^+^ T cells, CD8^+^CD127^+^ T cells, and CD8^+^KLRG1^+^ T cells in post-NAC tumor tissue were significantly associated with longer DFS (Fig. 6a–d), whereas higher CD20^+^ B cells, CD4^+^ T cells, CD8^+^ T cells, CD8^+^CD127^+^ T cells and CD8^+^KLRG1^+^ T cells in post-NAPC tumor tissue were significantly associated with longer DFS (Fig. 6e–i), which was consistent with the previous study in which the improved immune cell infiltration after treatment was associated with better survival in advanced NSCLC^31^. Collectively, these findings highlighted the potential utility of these subpopulations in predicting outcomes and suggested that B cells and CD8^+^ T cells may be key executors in mediating anti-PD-1-based therapies^29,32^.

### GEO dataset validates memory and effector CD8 signatures associated with therapeutic response and prognosis

**Fig. 7:**
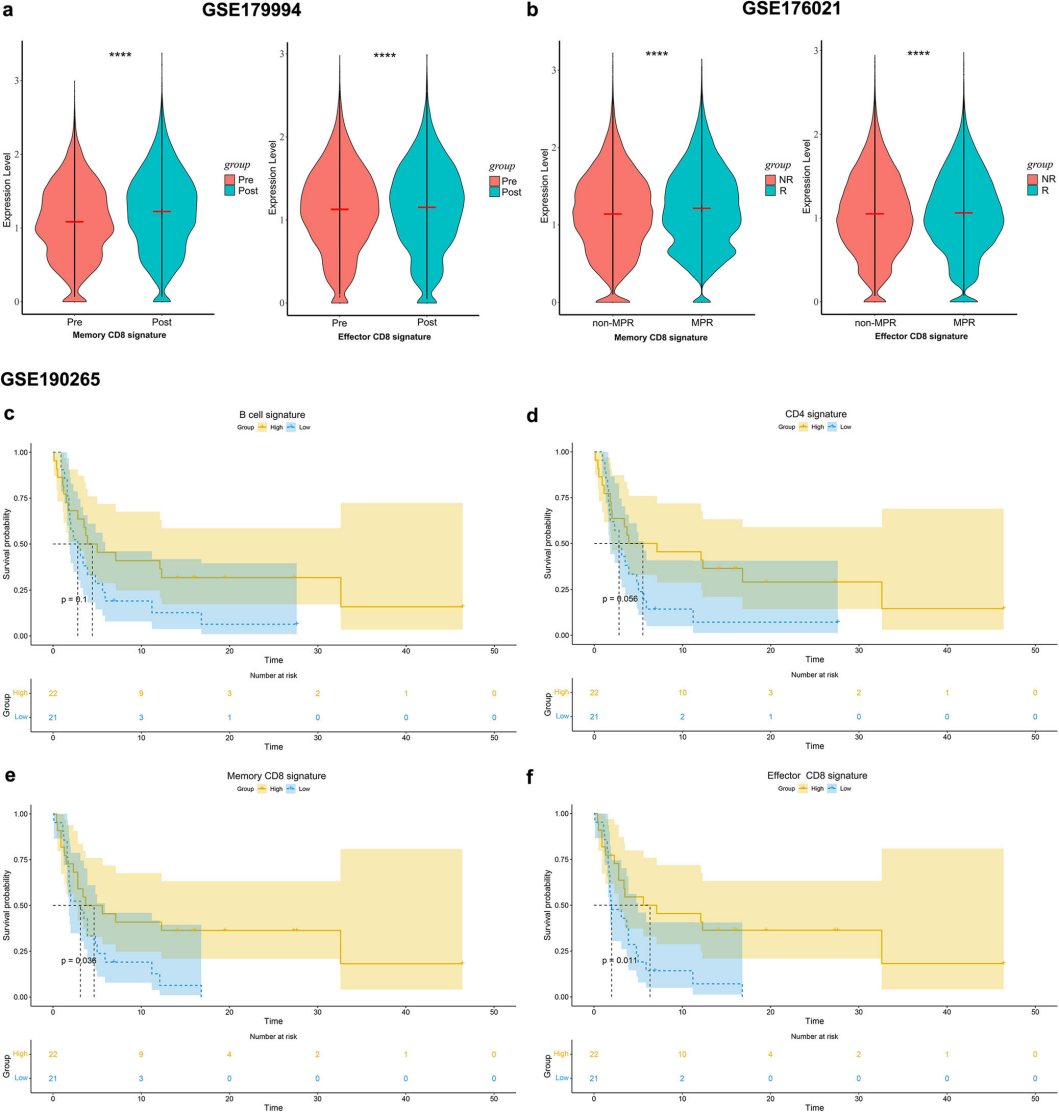
Memory and effector CD8^+^ T cell signatures associated with therapeutic response and prognosis. **a** The abundance of memory and effector CD8 signatures before and after PD-1 blockade plus chemotherapy in NSCLC dataset (GSE179994). Data are presented as mean. **b** The abundance of memory and effector CD8 signatures in non-MPR and MPR after neoadjuvant nivolumab for resectable NSCLC (GSE176021). Data are presented as mean. **c**–**f** Kaplan–Meier estimates of progression-free survival (PFS) for B-cell signature, CD4 signature, as well as memory and effector CD8 signatures in NSCLC patients receiving PD-1 blockade monotherapy (GSE190265). Patients were classified into highly infiltrated patients (ES ≥ median value) and low infiltrated patients (ES < median value) according to median ES in survival analysis.

To further confirm our findings, NSCLC databases treated with PD-1 blockade and with transcriptome sequencing data were searched in the GEO dataset, and three databases were included: GSE179994^33^, GSE176021^34^ and GSE190265^35^. The description of these datasets and the predefined gene sets of memory and effector CD8 signatures can be found in the Methods. Analysis of scRNA-seq data from paired pre- and post-treatment tumor tissues in advanced NSCLC patients receiving chemotherapy plus pembrolizumab in the GSE179994 dataset confirmed a higher enrichment of memory and effector CD8 signatures in post-treatment tumor tissues, in line with our scRNA-seq results (Fig. 7a). ScRNA-seq profiles of surgical tumor specimens obtained from the first-in-human clinical trial of neoadjuvant nivolumab in resectable NSCLC (GSE176021) also demonstrated that memory and effector CD8 signatures were more enriched in tumor tissues that acquired MPR (Fig. 7b). We analyzed the relationship between the immune cell infiltration level and progression-free survival (PFS) in 43 advanced NSCLC patients treated with anti-PD-1 antibody monotherapy in the GSE190265 dataset by gene set variation analysis (GSVA) and showed that higher B cell signature and CD4 signature correlated with better PFS (Fig. 7c, d), and higher memory and effector CD8 signatures in tumors were significantly associated with longer PFS in patients (Fig. 7e, f). Validation results from the GEO dataset are consistent with our scRNA results, and support that PD-1 blockade leads to an increase in memory and effector CD8^+^ T cells, this increase is positively correlated with therapeutic response and clinical outcome, and further confirming that the skewing of CD8^+^ T cells toward memory and effector phenotypes is a key determinant contributing to the improved benefit of anti-PD-1-based therapies.

## Discussion

The combination of neoadjuvant PD-1 blockade and chemotherapy has achieved unprecedented clinical success compared to neoadjuvant chemotherapy in terms of improved MPR rate, pCR rate, prolonged DFS and OS^5,6^. Several studies have explored the immunological impact of neoadjuvant chemotherapy on the TME in ovarian carcinoma^36^, breast cancer^37^, esophageal squamous cell carcinoma^38^ and pancreatic cancer^39^. Liu et al.^33^ revealed that clonal revival and expansion of precursor-exhausted T cells occurred during the combination of PD-1 blockade with chemotherapy in advanced lung cancer by scRNA-seq. By comparing pre- and post-treatment tumors from patients with resectable NSCLC treated with NAC or NAPC, we were able to further address the underlying mechanisms by which PD-1 blockade augments the efficacy of chemotherapy.

**Fig. 8:**
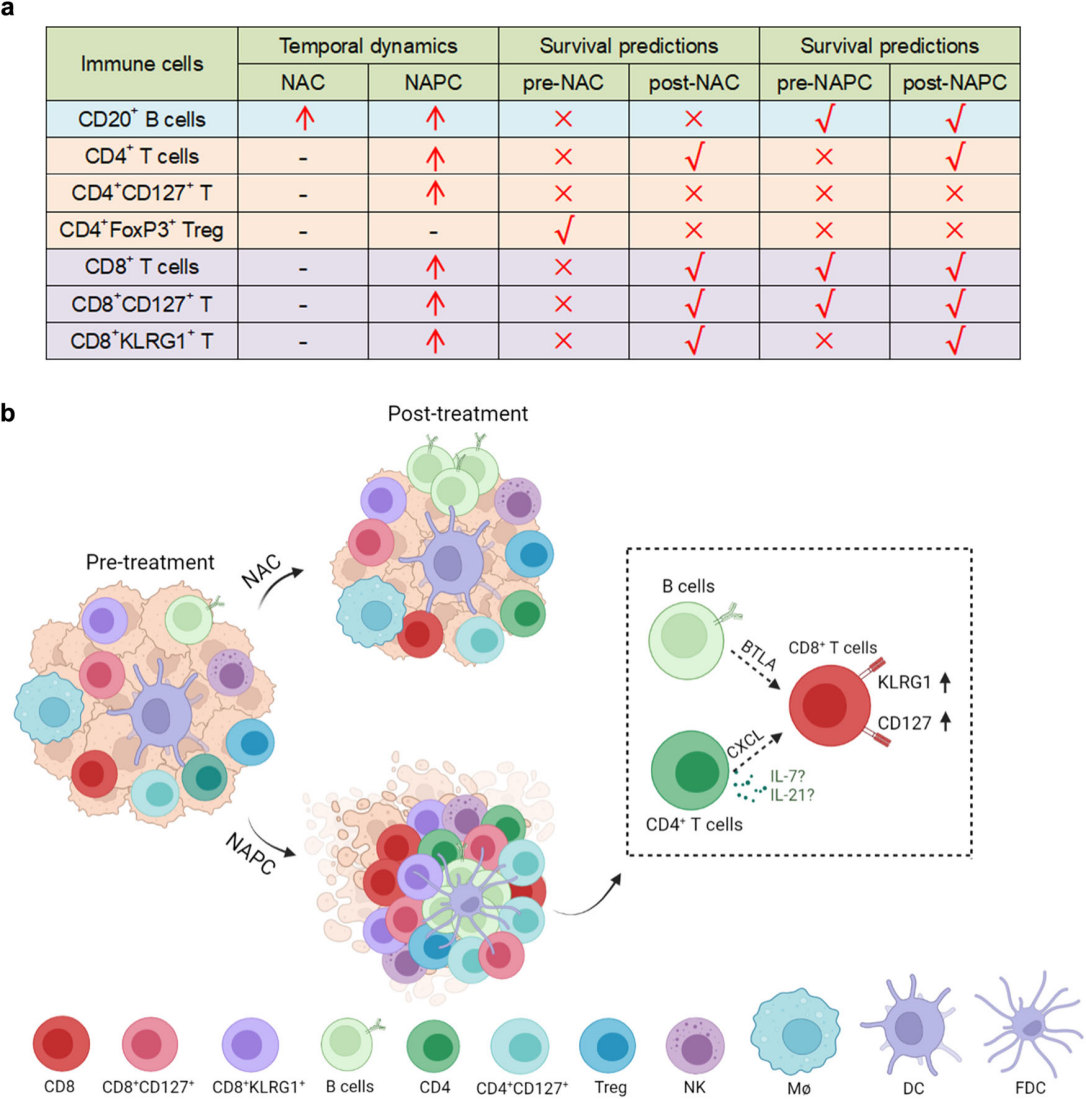
Summary of immune cell phenotypes and dynamics before and after NAC and NAPC therapy. **a** Temporal dynamics and survival predictions of key immune cell subsets following different treatments. Red arrows indicate elevated post-treatment cell ratios, right symbols indicate cell ratios with survival predictive value, and wrong symbols indicate cell ratios with no survival predictive value. **b** Immune features in NAC and NAPC tumors and their dynamics following different treatment regimens (Created with BioRender.com). NAC resulted in an increase only of CD20^+^ B cells, whereas NAPC promoted synergistic increases in CD20^+^ B cells, CD4^+^ T cells, CD4^+^CD127^+^ T cells, CD8^+^ T cells, CD8^+^CD127^+^ T cells, and CD8^+^KLRG1^+^ T cells. Treg cells did not differ before and after treatment in both NAC and NAPC groups. CD4^+^ T and CD20^+^ B cells may recruit CD8 to neighborhood via the CXCL signaling pathway and promote CD8^+^ T cells shifted toward CD127^+^ and KLRG1^+^ phenotypes via cytokines (such as IL-7, IL-21) or receptor-ligand interactions.

Our study elucidated the profound effect of PD-1 blockade in combination with chemotherapy versus chemotherapy alone on the neoadjuvant settings of resectable NSCLC patients. We confirmed through a multi-omics study that the addition of PD-1 blockade to chemotherapy resulted in a significant increase in CD20^+^ B cells, CD4^+^ T cells, CD8^+^ T cells, CD8^+^CD127^+^ T cells, and CD8^+^KLRG1^+^ T cells, which are key determinants of the efficacy of PD-1 blockade and correlated with patient survival. Spatial distribution patterns showed that CD8^+^ T cells, CD8^+^CD127^+^ T cells, and CD8^+^KLRG1^+^ T cells are in closer proximity to CD4^+^ T/CD20^+^ B cells in the NAPC group, indicating the shift of CD8^+^ T cells to CD127^+^ and KLRG1^+^ phenotypes required the assistance of CD4^+^ T and B cells (Fig. 8). Collectively, our study revealed the potential mechanism by which PD-1 blockade enhanced chemotherapy response and provides new ideas to improve the efficacy of combined immunotherapy and chemotherapy.

The outcome of ICBs therapy in cancer patients is related to the quality and magnitude of the T cell, NK and B cell responses in the TME^28,40^. We identified a pronounced increase in CD8^+^ T cells and their CD127^+^/KLRG1^+^ subsets after adding PD-1 blockade to neoadjuvant chemotherapy, which is in agreement with other previous studies showing that anti-PD-1 predominantly affects CD8^+^ T cells and that even a single dose of PD-1 blockade was able to induce rapid and robust CD8^+^ T cells expansion^41–43^. Moreover, not only the increase of TILs, but also the differentiation state and spatial distribution of TILs have been shown to determine clinical outcomes^44,45^. We noticed that TLS density was slightly higher in the post-NAPC, and the TLS morphology was different between post-NAC and post-NAPC. The TLSs in the post-NAC samples were smaller and mostly located in the periphery of the tumor, which presented like lymphoid aggregates, while the TLSs in the post-NAPC group were mature, organized TLSs with defined T cell/B cell zones (Fig. 5b). Tumor-infiltrating CD8^+^ T cells are comprised of heterogeneous subpopulations with distinct phenotype and functional state. CD8^+^ T cells expressing KLRG1 exhibit an enhanced ability to produce cytotoxic cytokines/granules, whereas CD8^+^ T cells express CD127 (IL7Rα) markers that fit a classic long-lived memory phenotype^46,47^. Generally CD8^+^KLRG1^+^ T cells are increased under inflammatory conditions and the ligand of KLRG1 has been described to be E-cadherin, N-cadherin, and R-cadherin expressed on epithelial and mesenchymal cells^48^. KLRG1 expression is tied to antigen-experience and aligned with cytotoxic T and NK cell differentiation (Supplementary Fig. 4), and up-regulated in human tumor samples after a variety of therapies that resulted in T cell proliferation, potentially contributing to adaptive resistance^49^. Tata A. et al.^50^ demonstrated the combination blockade of KLRG1 and PD-1 promotes immune control of local and disseminated cancers. A multiplexed single-cell analysis study reveals KLRG1^+^ cytotoxic T cells were enriched in colorectal cancer patients with good outcomes^51^. Based on these, we speculated that up-regulation of the checkpoint receptor KLRG1 may be a compensatory inhibitory pathway to anti-PD-1 induced T cell activation. Future studies will need to elucidate the role of KLRG1 as a potential resistance mechanism and determine whether KLRG1 can be targeted to improve anti-tumor responses in patients.

Although anti-PD1 antibodies primarily target T cells, B and CD4^+^ T cells were essential in ICB-driven anti-tumor responses via secreting antibody and helping T cell response, as indicated by depletion of B cells significantly reduced therapeutic response to anti-PD1 in mice model^52^. CD4^+^ T cells can profoundly modulate the TME by secreting different types of cytokines^53^ or by directly eliminating cancer cells^54^. Cui et al.^20^ demonstrated that neoantigen-driven B cells promoted CD4^+^ T follicular helper cells to secret IL-21 to boost anti-tumor CD8^+^ T cell responses in a murine model of lung adenocarcinoma. Consistent with previous results, we noted synergistic increase in CD20^+^ B cells, CD4^+^ T cells, and CD8^+^ T cells in the TME after NAPC therapy. The increase in CD20^+^ B cells and CD4^+^ T cells was positively correlated with CD127^+^ and KLRG1^+^ CD8 T cells. Spatial distribution analysis confirmed potential cell-cell interaction between CD20^+^ B/CD4^+^ T cells and CD8^+^ T cells in the NAPC group. In contrast, in the NAC group, although the proportion of CD20^+^ B cells increased significantly after treatment, the therapeutic effect was limited due to the absence of a synergistic increase of CD4^+^ and CD8^+^ T cells. This suggests that individual lymphocyte subsets are not solely responsible for tumor immune control and that the proper location, aggregation, interplay and co-stimulation of all lymphocyte subsets are required for a successful antitumor immune response^22^. We also predicted the specific ligand-receptor pairs for CD4^+^ T or B cells interacting with CD8^+^ T cells by CellChat^25^ and found that CD4^+^ T and B cells may recruit and regulate CD8^+^ T cells function through CXCL and cytokines, and the involved pathways will be validated by our subsequent studies. Taken together, we found that T and B cells synergy increased after NAPC, and that CD4^+^ T cells may collaborate with B cells to promote CD8^+^ T cells phenotypic changes to CD127^+^ and KLRG1^+^ CD8 T cells, indicating that these subsets may be central elements by which PD-1 blockade enhanced anti-tumor immunity.

Despite research suggesting that preexisting tumor-infiltrating immune cells are associated with favorable clinical outcomes and response to immunotherapy^13,55^, a recent study illustrated that assessing the immune profile of tumor biopsies in the early course of ICB therapy was a better predictor of response than assessing pretreatment samples^56^. We quantified TILs of different subsets and phenotypes before and after neoadjuvant therapy in the NAC and NAPC groups by mIHC and analyzed their prognostic role on patient survival. We demonstrated that pre-treatment TILs predicted patient survival and post-treatment TILs correlated with clinical outcome and highlighted the important role of B cells and CD8^+^ T cells in mediating anti-tumor immune responses during PD-1 blockades therapy, supporting the potential for novel therapies directed at B cells and CD8^+^ T cells^29,32^.

In conclusion, we performed a comprehensive assessment of tumor immune status before and after NAC and NAPC treatment using scRNA-seq, mIHC and GSE database validation, and analyzed altered immune cell infiltration levels, phenotypes, and interactions in patients with resectable NSCLC after adding PD-1 blockade to neoadjuvant chemotherapy, and demonstrated that anti-PD1 therapy could reshape the tumor immune microenvironment based on chemotherapy-induced alterations. Our findings shed light on the underlying mechanism by which ICBs augment chemotherapy response. We identified potentially novel candidate T cell subsets that may be therapeutically targeted to improve upon existing immunotherapies for NSCLC. Our study has some limitations which should be taken into consideration when interpreting the data. First, patient inclusion was limited by specimen availability, with only one NAC used for scRNA-seq and a relatively small number of pre-treatment biopsies. This may have resulted in some findings trending toward a difference but not reach statistical significance. There is a need to extend these findings to a larger cohort. Second, the median follow-up time differed between the two groups due to our exploring the feasibility of PD-1 blockade combined with chemotherapy as a neoadjuvant treatment option later in the clinical setting than chemotherapy. Studies with longer follow-up are needed to clarify the association of TILs with survival.

## Methods

### Patients and study design

Sixty-five patients with resectable NSCLC who received two cycles of neoadjuvant therapy prior to surgical resection were included in this study, 30 of whom were treated with NAC and 35 with NAPC. The included patients received neoadjuvant paclitaxel 175 mg/m^2^ plus carboplatin (area under curve 5; 5 mg/mL per min) for squamous cell carcinoma and pemetrexed 500 mg/m^2^ plus carboplatin for adenocarcinoma, with or without intravenous pembrolizumab 200 mg on day 1, 21 days each cycle, for two cycles before surgical resection, and then followed by two cycles after surgical resection.

Eligible patients were aged 18 years or older and had histologically treatment-naive and surgically resectable IIA–IIIB NSCLC (American Joint Committee on Cancer 7^th^ edition criteria). All patients had an Eastern Cooperative Oncology Group performance status of 0 or 1, adequate organ function, and adequate pulmonary function. Patients were excluded from enrollment if they had a known EGFR mutation or ALK translocation; a history of autoimmune disease, interstitial lung disease or prior cancer; had interstitial lung disease or pneumonitis; receiving ongoing glucocorticoid or immunosuppressant therapy; previously treated with checkpoint inhibitors or other drug that target T-cell co-stimulation or immune checkpoint pathways. Patients with acute or chronic hepatitis virus infection or active tuberculosis were also excluded.

Clinicopathological parameters, including age, sex, smoking history, cTNM stage, pathological type, neoadjuvant and adjuvant therapy regimens, type of resection, pathological response, recurrence or not, and follow-up time were obtained (Table 1). The last follow-up was conducted on September 30, 2022, with a median follow-up time of 94 months for NAC and 35 months for NAPC. Disease-free survival (DFS) was defined as the time from the date of surgery to the date of recurrence or last follow-up. Overall survival (OS) was defined as time from the date of surgery to the date of any caused death or last follow-up. The study was approved by the Ethics Committee of Tianjin Cancer Institute & Hospital and all patients enrolled in the study provided written informed consent.

### Pathologic assessment of response to treatment

Pathological response to neoadjuvant therapy was assessed independently by two pathologists after tumor resection according to previously described methods^57,58^. Briefly, all hematoxylin and eosin-stained (HE) slides from each patient were reviewed and the tissue was divided into three components: viable tumor area, necrosis, and stromal tissue, including fibrosis and inflammatory cells. The percentage of viable tumor area to total tissue area on each slide was estimated and the average taken as the patients’ final response rate. The number of slides examined per patient ranged from 1 to 5, with an average of 3.3 per case. MPR was defined as 10% or less viable tumor cells, and pCR was defined as the absence of viable tumor cells.

### Single-cell RNA sequencing

Surgically resected fresh tumor tissue from six patients treated with two cycles of NAPC and one patient treated with two cycles of NAC was cut into pieces of approximately 1–3 mm³ (Clinicopathological information, cell counts and response to neoadjuvant therapy for single-cell sequencing patients are shown in supplementary Table 1). Then, the pieces were transferred to the gentle MACS C Tubes (Miltenyi Biotec, Bergisch Gladbach, Germany), with 5 mL of digestive enzyme included in the Tumor Dissociation Kit (Miltenyi Biotec, Bergisch Gladbach, Germany). Then the tissues were made into single-cell suspension using the gentleMACS Dissociator (Miltenyi Biotec, Bergisch Gladbach, Germany) for 60 min on a rotor at 37°C according to the manufacturer’s instructions. After removing of red blood cells using erythrocyte lysis solution (Miltenyi Biotec, Bergisch Gladbach, Germany), the cells were filtered using a 40 µm strainer (BD Falcon, New York, USA). CD45^+^ cells were sorted by using the Human CD45 Microbeads Isolation Kit (Miltenyi Biotec, Bergisch Gladbach, Germany). 10X Chromium Single cell 5′ and human variable, diversity, and joining (VDJ) library construction (10x Genomics) were used to prepare the libraries according to the manufacturer’s instructions. All subsequent steps were performed following the standard manufacturer protocols. Purified libraries were analyzed using an Illumina Hiseq X Ten sequencer (Illumina, San Diego, CA, USA) with 150-base pair (bp) paired-end reads.

### Dimension reduction and unsupervised clustering for scRNA-seq data

Cell Ranger 3.1.0 was used to align and quantify the generated scRNA-seq data against the GRCh38 human reference genome. After generating the gene expression matrix, we filtered the cell-identifying barcodes to avoid dead cells and other artifacts with Seurat 3.2.1. Specifically, we filter out cells with less than 200 or more than 6000 genes detected per cell, or with less than 1000 UMI, or with more than 10% mitochondrial genes. Principal component analysis (PCA) was performed on the variable gene matrix to reduce noise, and the top 50 components were used for downstream analyses. Unsupervised clustering of cells was performed using the Seurat package based on gene expression profiles and passed to UMAP for visualization of clusters. Batching effect between samples was corrected by “FindIntegrationAnchors” (MNN algorithm) function in the Seurat. We tested the resolution of the “FindClusters” from 0.2-1.0, and found that 0.8 was the most appropriate resolution. Clusters were identified and annotated according to canonical immune cell markers. DEGs were identified by the “FindMarkers” and “FindAllMarkers” in Seurat, using the following criteria: Log2FC>0.25, p < 0.05, min.pct>0.1. Go biological process enrichment analysis of DEGs was performed by the Clusterprofiler package (v.3.14.3). Cell-cell communications were analyzed using CellChat 1.1.3 (https://github.com/sqjin/CellChat)^25^.

### GSEA analysis

The signatures for B-cell, CD4 and CD8 subpopulations were derived from previous published studies^13,15^ and CellMarker (http://bio-bigdata.hrbmu.edu.cn/CellMarker)^59^. GSEA analysis was performed on the signature gene sets of B-cell, CD4 and CD8 subpopulations in NAC and NAPC, and heat maps were drawn according to the enrichment score results. The genes included in each cell signature are listed in Supplementary Table 2.

### AddModuleScore analysis

To illustrate the functional properties of CD8^+^ T cells and each CD8 cluster, we collected sets of genes from the literature and calculated the functional scores of each cell using the “AddModuleScore” in Seurat^18^. The definitions of exhaustion-related, naïve-related, cytotoxic and inflammatory genes of T cells were derived from Zhang et al.^17^ The genes for each score are listed in supplementary Table 3.

### PD-L1 immunohistochemistry staining

Immunohistochemistry (IHC) staining for PD-L1 was performed in the pathology department using monoclonal mouse anti-human PD-L1 clone 22C3 (LOT10145059, Dako, Carpentaria, CA, USA) and interpreted by a pathologist. The tumor proportion score (TPS) and combined positive score (CPS) were calculated as the percentage of at least 100 viable cells with complete or partial membrane staining. For patients without residual tumor after treatment only CPS was calculated.

### mIHC staining and multispectral analysis

FFPE tumor tissue samples were collected both at initial biopsy when diagnosed before neoadjuvant therapy (pre-treatment) and at surgery after neoadjuvant therapy (post-treatment). Due to the unavailability of some biopsy specimens, we obtained 16 paired pre-NAC and post-NAC samples and 14 unpaired post-NAC samples in the NAC group, and 18 paired pre-NAPC and post-NAPC samples, 5 unpaired pre-NAPC and 12 unpaired post-NAPC samples in the NAPC group. MIHC was conducted in FFPE slides using PANO 7-plex IHC kit (Panovue, Beijing, China). After dewaxing and rehydration, slides were prepared for antigen retrieval by microwave heating for 15 min in Tris-EDTA buffer (pH 9.0). The tissue was blocked using blocking solution for 10 min, and incubated with the 1^st^ primary antibody overnight in a 4°Crefrigerator. Then the tissue was incubated with horseradish peroxidase-conjugated secondary antibody and tyramide signal amplification (TSA) for 10 min each. The slides were microwave treated after TSA operation to remove the Ab-TSA complex. Repeated the above procedure for the incubation of 2^nd^ primary antibody until all primary antibodies was labeled, and finally the nucleus was stained with 4′-6′- diamidino-2-phenylindole (DAPI). Primary antibodies information used for mIHC staining was listed in Supplementary Table 8.

Mantra multispectral imaging system was used to scan and photograph multispectral images at 20× magnification with the same exposure times. Ten randomly fields in each stained slides were selected. The multispectral images were split and segmented using InForm image analysis software. Individual cells were identified with a nuclear segmentation algorithm using DAPI staining, together with a cellular mask around each nucleus to quantitate surface marker expression for each cell. The ratio of positive cell counts to DAPI was used to verify differences in TILs.

### TLS density quantification

TLSs were identified and quantified using immunofluorescence staining for CD20, CD4 and CD8. TLSs were identified as aggregates of lymphocytes having histological features with analogous structures to that of lymphoid tissue with germinal centers (including B cells (CD20) and T cells (CD4/CD8), appearing in the tumor area. TLS density is defined as the total number of structures identified either within the tumoral area or in direct contact with the tumoral cells on the margin of the tumors (numbers of TLS per mm^2^ area).

### Spatial distribution patterns of TILs

Spatial distribution among TILs was analyzed with R software (Version 3.1.0). We used a bivariate point pattern characterized by the bivariate K(r) functions to represent the relative spatial distributions of TILs^22^. Each cell of the same phenotype was used as a reference cell and the number of other cells within a given radius was calculated. We performed spatial distance analysis for r = 10 μm, 20 μm, 30 μm, 40 μm, 60 μm, and 80 μm, respectively, and found consistent trends for different distances. Based on previous studies^23,60^, the proximity distance was defined as the average number of cells distributed within a 30um radius from the nuclear center of each reference cell.

The bivariate K(r) function is defined as the expected number of cells appearing within the radius and the formula is:

$$K(r)={(\alpha /({n}^{\ast }(n-1)))}^{\ast }sum[i,j]I(d[i,j]\le r)e[i,j]$$

α in the formula is the area, n is the number of cells, d [i, j] is the distance between two cells i and j, and I (d [i, j] ≤ r) is the logical decision function within a given radius *r*.

The Euclidean distance between two points i = (ix, iy) and j = (jx, jy) is defined as follows:

$$\parallel i-j\parallel ={[{(ix-jx)}^{2}+{(iy-jy)}^{2}]}^{1/2}$$

### GEO dataset validation

NSCLC datasets treated with PD-1 blockade and with transcriptome sequencing data were searched in the GEO dataset, and three databases were included: GSE179994 (scRNA-seq of pre- and post-treatment tumor biospies from patients with advanced NSCLC after PD-1 blockade in combination with chemotherapy)^33^, GSE176021 (scRNA-seq of peripheral blood and surgical tumor specimens after neoadjuvant nivolumab for resectable NSCLC)^34^, and GSE190265 (bulk RNA-seq of tumor tissues with survival information in NSCLC patients receiving first- or second-line PD-1 blockade monotherapy)^35^. We downloaded the raw sequencing data and processed expression matrices, filtered out peripheral blood and nontumor tissue sequencing data, calculated the relative infiltration abundance of lymphocyte signatures in each sample by GSVA based on predefined gene sets, and represented the relative infiltration abundance by enrichment score (ES). Patients were classified into highly infiltrated patients (ES ≥ median value) and low infiltrated patients (ES < median value) according to median ES in survival analysis. B-cell, CD4 and CD8 subset signatures are defined as in the GSEA analysis (Supplementary Table 2).

### Statistical analysis

Statistical analyses were performed with SPSS 24.0 and Graphpad prism 8.0 software. Chi-square tests were used to compare two groups of categorical variables. The immune cells density was described as the mean ± standard deviation (SD). Comparison of unpaired differences between two groups was assessed by Mann-Whitney test, and paired numerical variables between two groups were assessed by Wilcoxon matched-pairs signed rank test. Comparisons of numerical variables among more than two groups were assessed by Kruskal-Wallis test. Two-way ANOVA with multiple comparisons was used to compare the difference between pre to post, non-MPR versus MPR. The optimal cutoff values for classifying patients into high and low groups were generated by the “survminer” package of the R software. Survival analysis was performed using the Kaplan-Meier method, and group comparisons were conducted by log-rank test. Correlation analysis was performed using the Spearman’s rank method. All P values were two-sided and less than 0.05 is considered to be statistically significant (* p < 0.05, ** p < 0.01, *** p < 0.001, **** p < 0.0001, ns: not significant).

### Reporting summary

Further information on research design is available in the Nature Research Reporting Summary linked to this article.

## Supplementary information

Supplementary File

REPORTING SUMMARY

## Data Availability

Single-cell transcriptome data in this study has been deposited in the Gene expression Omnibus (GEO) dataset (GSE229353). GSE179994, GSE176021 and GSE190265 for validation are available in the GEO dataset.

## Code availability

The codes that support the findings of this study are available from the corresponding authors on reasonable request.
