# Supplementary material for: PD-1 blockade potentiates neoadjuvant chemotherapy in NSCLC via increasing CD127+ and KLRG1+ CD8 T cells
Source: NPJ Precis Oncol. 2023 May 25;7:48. doi: 10.1038/s41698-023-00384-x (PMC10213055; doi:10.1038/s41698-023-00384-x)
Supplement: Supplementary file 1 — Supplementary File [file 41698_2023_384_MOESM1_ESM.pdf]

Supplementary Table 1. Clinicopathological information, cell counts and response to neoadjuvant therapy for single-cell sequencing patients.

Patient	Age	Sex	Therapy	Cell counts	Pathology	RVT%	Pathologic response
P01	68	Male	NAC	4057	SCC	40%	non-MPR
P02	69	Male	NAPC	3045	SCC	90%	non-MPR
P03	51	Female	NAPC	2182	SCC	0%	MPR
P04	66	Female	NAPC	2888	AD	80%	non-MPR
P05	67	Male	NAPC	4474	SCC	0%	MPR
P06	62	Male	NAPC	7579	SCC	0%	MPR
P07	46	Male	NAPC	2636	AD	60%	non-MPR

RVT, residual viable tumor cells; SCC, squamous cell carcinoma; AD, adenocarcinoma; MPR, major pathological response; NAC, neoadjuvant chemotherapy; NAPC, neoadjuvant pembrolizumab and chemotherapy.

Supplementary Table 2. Signature genes of B cell, CD4 and CD8 subpopulations in GSEA analysis.

Cell types	Genes included in signature
B cells	CD19, MS4A1, CD79A, CD79B
Naïve $CD4^+$ T cells	CD4, LEF1, TCF7
Memory $CD4^+$ T cells	CD4, CD27, CCR7, IL7R
Treg cells	CD4, IL2RA, FOXP3, CCR8, LAYN, CTLA4, TNFRSF9, TNFRSF18, MAGEH1, IKZF2
Naïve $CD8^+$ T cells	CD8A, CD8B, CD45RA, LEF1, TCF7
Memory $CD8^+$ T cells	CD8A, CD8B, CD45RO, CD27, CD62L, CCR7, IL7R
Effector $CD8^+$ T cells	CD8A, CD8B, KLRG1, IFNG, GZMK, GZMB, PRF1, TBX21
Exhausted $CD8^+$ T cells	CD8A, CD8B, PDCD1, CTLA4, TOX, HAVCR2, ENTPD1, LAYN, LAG3
Proliferating $CD8^+$ T cells	CD8A, CD8B, STMN1, TUBB, MKI67

Supplementary Table 3. Signature genes used to define functional status in T cells.

naïve	exhaustion	cytotoxic	inflammatory	effector	regulatory
CCR7	HAVCR2	PRF1	IRF1	GZMA	TNFRSF9
TCF7	CXCL13	IFNG	CD8A	GZMB	FOXP3
LEF1	CCL3	GNLY	CCL2	PRF1	CTLA4
SELL	SIRPG	NKG7	CCL3	EOMES	CCR8
	IFNG	GZMB	CCL4	IFNG	ADORA2A
	TIGIT	GZMA	CXCL9	TNF	REL
	GZMB	GZMH	CXCL10	CXCL9	TGFB1
	PDCD1	KLRK1	ICOS	CXCL10	HELIOS
	PARK7	KLRB1	GZMK	CD8A	
	TNFRSF9	KLRD1	HLA-DMA	CD4	
	ACP5	CTSW	HLA-DMB	FOXP3	
	CTLA4	CST7	HLA-DOA	ICOS	
	RBPJ		HLA-DOB	CTLA4	
	MIR155				
	CXCR6				
	CD27				
	FKBP1A				
	BST2				
	TPI1				
	MIR155HG				
	PTTG1				
	CD63				
	SAMSN1				
	RGS1				
	CD27-AS1				
	ITGAE				
	MIR4632				
	HLA-DRA				
	IGFLR1				
	KRT86				
	ENTPD1				
	DUSP4				
	SIT1				
	TOX				
	PHLDA1				
	CCND2				
	GPR25				
	LAYN				
	PRDX5				

	SARDH				
	FASLG				
	MIR3917				
	ANXA5				
	CTSD				
	PDIA6				
	RANBP1				
	FKBP1A-SDCBP2				
	COTL1				
	TNFRSF1B				
	IDH2				
	CD38				
	CD82				
	LAG3				
	MIR497HG				
	APOBEC3C				
	ITM2A				
	COX5A				
	IFI35				
	NDFIP2				
	TNFRSF18				
	KRT81				
	DNPH1				
	RGS2				
	HMGN1				
	DYNLL1				
	SNRPB				
	STRA13				
	SYNGR2				
	RAB27A				
	PSMC3				
	GALM				
	FABP5				
	UBE2L6				
	MYO7A				
	PRDX3				
	DDIT4				
	STMN1				
	CDK2AP2				
	VCAM1				
	SNAP47				
	PSMB3				
	ISG15				

	HLA-DRB5				
	CKS2				
	TNIP3				
	CD7				
	PSMD4				
	ATP6V1C2				
	PSMD8				
	HLA-DRB6				

Supplementary Table 4. Spearman correlation of PD-L1 and TILs in pre-NAPC and post-NAPC.

1.Spearman correlation of PD-L1 and TILs in pre-NAPC.							
**Correlation**	PD-L1 vs. $CD4^+$ T cells	PD-L1 vs. $CD8^+$ T cells	PD-L1 vs. $CD20^+$ T cells	PD-L1 vs. $CD4^+CD127^+$ T cells	PD-L1 vs. $CD4^+FoxP3^+$ T cells	PD-L1 vs. $CD8^+CD127^+$ T cells	PD-L1 vs. $CD8^+KLRG1^+$ T cells
**Spearman r**							
r	0.4577	0.1972	0.325	0.2108	0.1882	0.09787	-0.13
**P value**							
P (two-tailed)	0.0561	0.4328	0.1882	0.4011	0.4547	0.6993	0.6071
P value summa	ns	ns	ns	ns	ns	ns	ns
Exact or appro	Approxima	Approxim	Approxim	Approximate	Approximate	Approximate	Approximate
Significant? (a	No	No	No	No	No	No	No

2.Spearman correlation of PD-L1 and TILs in post-NAPC.							
**Correlation**	PD-L1 vs. $CD4^+$ T cells	PD-L1 vs. $CD8^+$ T cells	PD-L1 vs. $CD20^+$ T cells	PD-L1 vs. $CD4^+CD127^+$ T cells	PD-L1 vs. $CD4^+FoxP3^+$ T cells	PD-L1 vs. $CD8^+CD127^+$ T cells	PD-L1 vs. $CD8^+KLRG1^+$ T cells
**Spearman r**							
r	-0.0729	-0.0577	0.0354	-0.1254	0.1698	-0.08522	-0.1048
**P value**							
P (two-tailed)	0.7236	0.7794	0.8637	0.5415	0.4071	0.6789	0.6104
P value summa	ns	ns	ns	ns	ns	ns	ns
Exact or appro	Approxima	Approxim	Approxim	Approximate	Approximate	Approximate	Approximate
Significant? (a	No	No	No	No	No	No	No

Supplementary Table 5. Spearman correlation of tumor size at surgery and TILs in NAC and NAPC.

1.Spearman correlation of tumor size at surgery and TILs in NAC.							
**Correlation**	Tumor size (cm) vs. $CD4^+$ T cells	Tumor size (cm) vs. $CD8^+$ T cells	Tumor size (cm) vs. $CD20^+$ T cells	Tumor size (cm) vs. $CD4^+CD127^+$ T cells	Tumor size (cm) vs. $CD4^+FoxP3^+$ T cells	Tumor size (cm) vs. $CD8^+CD127^+$ T cells	Tumor size (cm) vs. $CD8^+KLRG1^+$ T cells
**Spearman r**							
r	-0.3843	-0.0957	-0.1738	-0.3945	-0.4001	-0.2371	-0.3149
**P value**							
P (two-tailed)	0.036	0.6148	0.3584	0.031	0.0285	0.2071	0.0901
P value summa	*	ns	ns	*	*	ns	ns
Exact or appro	Approxima	Approxim	Approxim	Approximate	Approximate	Approximate	Approximate
Significant? (a	Yes	No	No	Yes	Yes	No	No

2.Spearman correlation of tumor size at surgery and TILs in NAPC.							
**Correlation**	Tumor size (cm) vs. $CD4^+$ T cells	Tumor size (cm) vs. $CD8^+$ T cells	Tumor size (cm) vs. $CD20^+$ T cells	Tumor size (cm) vs. $CD4^+CD127^+$ T cells	Tumor size (cm) vs. $CD4^+FoxP3^+$ T cells	Tumor size (cm) vs. $CD8^+CD127^+$ T cells	Tumor size (cm) vs. $CD8^+KLRG1^+$ T cells
**Spearman r**							
r	-0.1446	-0.1627	-0.3141	0.01558	-0.2608	0.0187	-0.1081
**P value**							
P (two-tailed)	0.4718	0.4175	0.1105	0.9385	0.1888	0.9262	0.5913
P value summa	ns	ns	ns	ns	ns	ns	ns
Exact or appro	Approxima	Approxim	Approxim	Approximate	Approximate	Approximate	Approximate
Significant? (a	No	No	No	No	No	No	No

Supplementary Table 6. Mean percentage of TILs before and after NAC with different chemotherapy regimens.

TILs	Pre-NAC			Post-NAC		
	PTX+CBP (N=12)	PEM+CBP (N=4)	p value	PTX+CBP (N=18)	PEM+CBP (N=12)	p value
$CD20^+$	0.7000	0.5400	0.7703	14.95	9.315	0.5732
$CD4^+$	8.795	10.05	0.6835	14.87	16.44	0.1236
$CD4^+CD127^+$	1.560	2.350	0.8615	2.104	3.898	0.0116
$CD4^+FOXP3^+$	0.8100	1.265	0.7703	1.754	2.252	0.3048
$CD8^+$	5.060	5.930	0.2016	8.300	6.495	0.2852
$CD8^+CD127^+$	0.2950	0.6600	0.7703	0.3443	0.4124	0.6021
$CD8^+KLRG1^+$	0.3650	0.1950	0.5429	0.6545	0.5059	0.3684

Data was presented with median, p values were determined by Mann-Whitney test. NAC, neoadjuvant chemotherapy; NAPC, neoadjuvant pembrolizumab and chemotherapy; TILs, tumor-infiltrating lymphocytes; PTX, paclitaxel; CBP, carboplatin; PEM, pemetrexed.

Supplementary Table 7. Mean percentage of TILs before and after NAPC with different chemotherapy regimens.

TILs	Pre-NAPC		p value	Post-NAPC		p value
	PTX+CBP (N=16)	PEM+CBP (N=7)		PTX+CBP (N=23)	PEM+CBP (N=7)	
$CD20^+$	0.6800	0.5500	0.6822	19.90	14.37	0.3107
$CD4^+$	7.760	9.410	0.2776	19.53	21.22	0.8480
$CD4^+CD127^+$	1.330	0.720	0.4510	5.013	6.273	0.8107
$CD4^+FOXP3^+$	0.5050	1.180	0.1918	1.905	1.560	0.5975
$CD8^+$	4.740	4.380	0.8195	15.78	11.72	0.3347
$CD8^+CD127^+$	0.2250	0.1600	0.6599	1.036	0.5444	0.1901
$CD8^+KLRG1^+$	0.1400	0.3000	0.4405	1.389	1.473	0.9618

Data was presented with median, p values were determined by Mann-Whitney test. NAC, neoadjuvant chemotherapy; NAPC, neoadjuvant pembrolizumab and chemotherapy; TILs, tumor-infiltrating lymphocytes; PTX, paclitaxel; CBP, carboplatin; PEM, pemetrexed.

Supplementary Table 8. Primary antibodies Information for mIHC staining of pre- and post-therapy FFPE slides from NSCLC patients treated with NAC or NAPC.

Antibodies	Source	Identifier	Diluted concentration	Fluorescent channel
Rabbit monoclonal anti-CD4 antibody	Abcam	ab133616	1:1000	PPD520
Rabbit monoclonal anti-CD127 antibody	Abcam	ab259806	1:1500	PPD540
Mouse monoclonal anti-FOXP3 antibody	Abcam	ab20034	1: 400	PPD570
Rabbit monoclonal anti-KLRG1 antibody	R&D	MAB70293-SP	1: 400	PPD620
Rabbit monoclonal anti-CD8 antibody	Abcam	Ab237710	1: 800	PPD650
Rabbit monoclonal anti-CD20 antibody	Abcam	ab78237	1:1000	PPD690

Supplementary Figures and legends

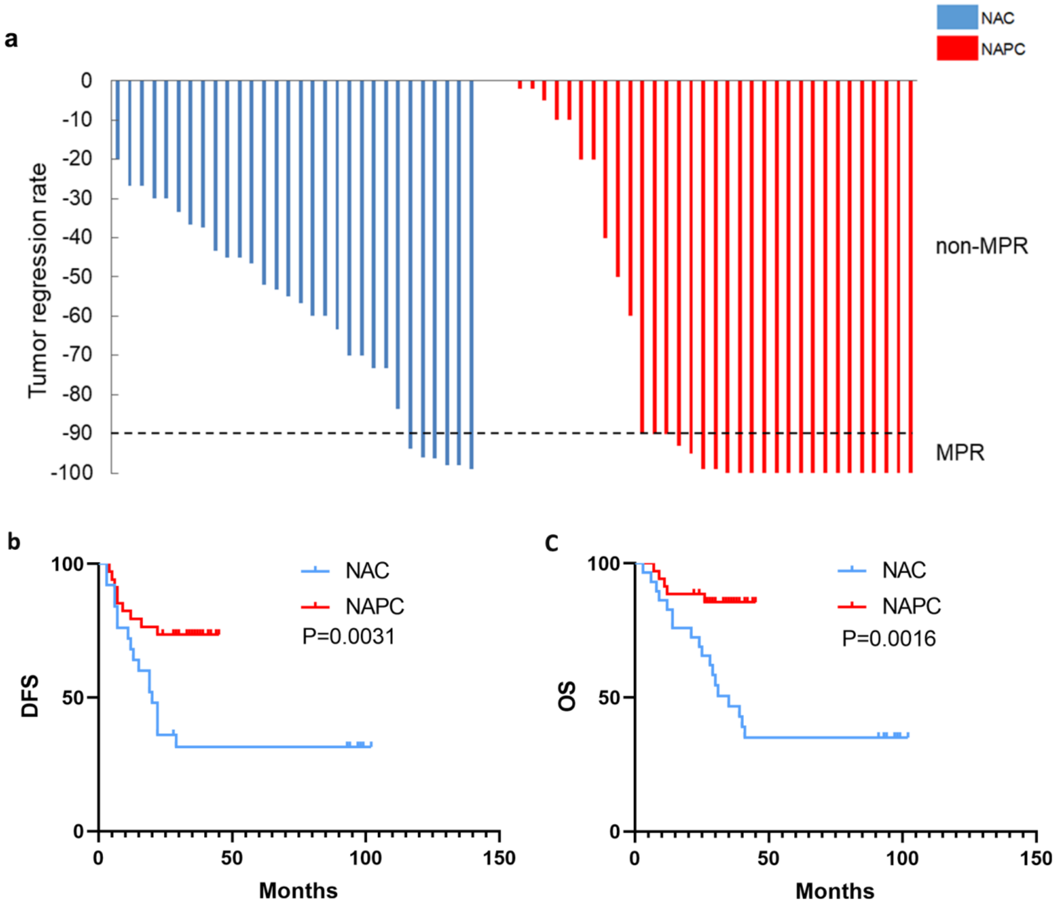

Supplementary Figure 1. Pathologic response and prognosis of NSCLC patients treated with NAC or NAPC therapy.

a Waterfall chart represented the pathological regression of each patient in two treatment regimens, with the horizontal dashed line indicating the threshold of MPR (90% regression). **b,c** Kaplan–Meier estimates of disease-free survival (DFS) and overall survival (OS) in the NAC and NAPC cohorts.

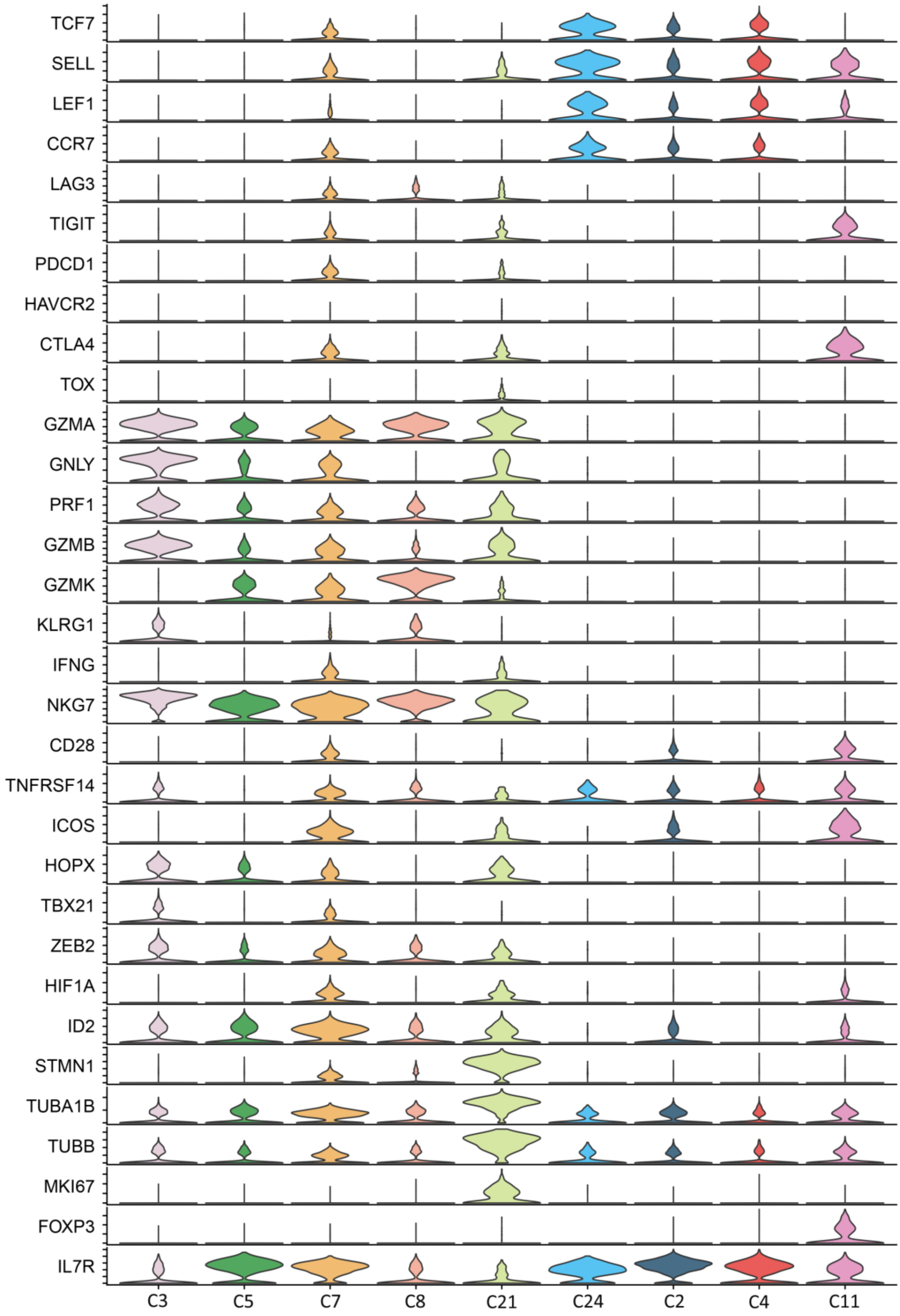

Supplementary Figure 2. Violin map of cluster signature genes of $CD8^+$ T-cell and $CD4^+$ T-cell clusters.

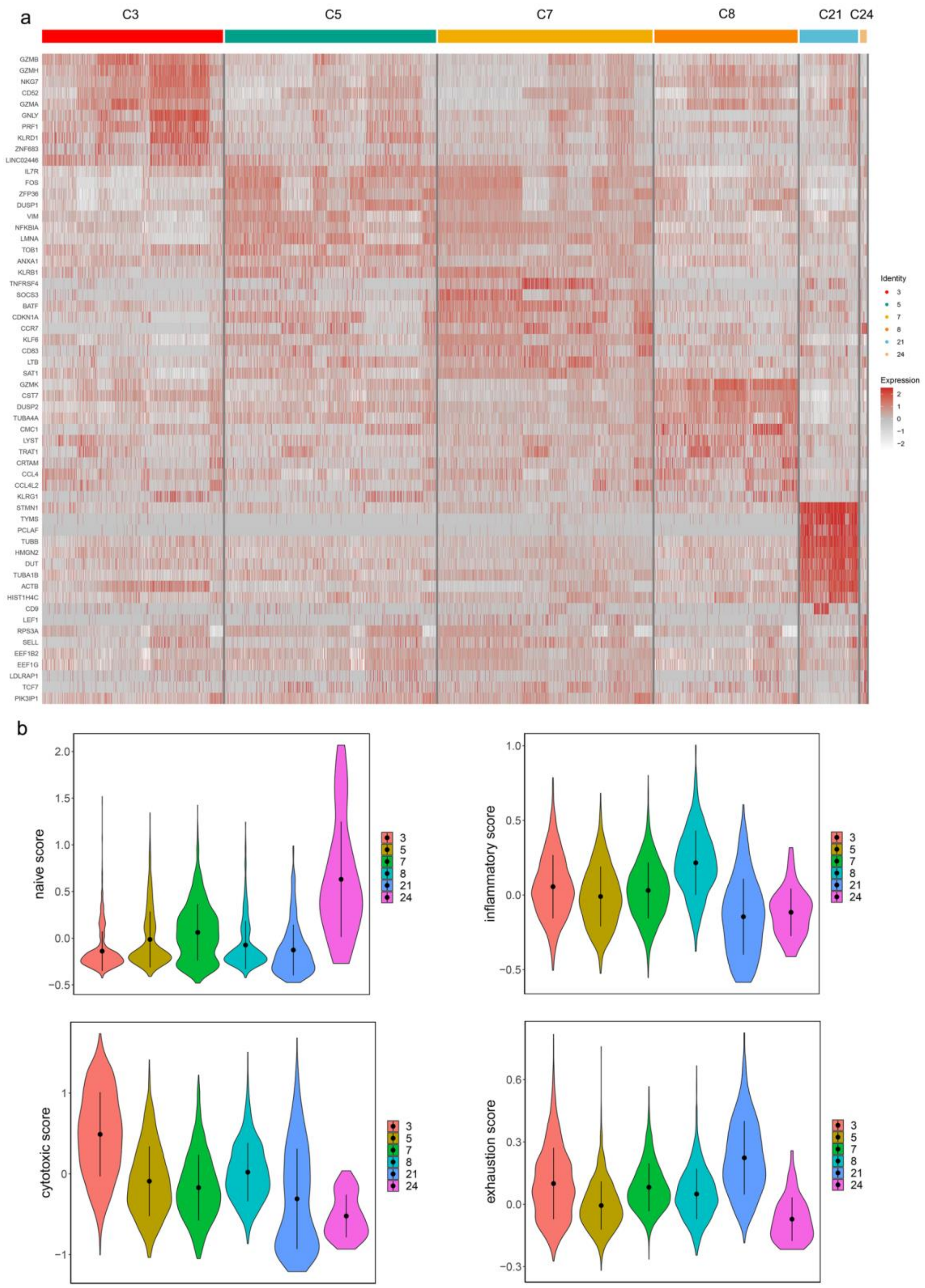

Supplementary Figure 3. Clustering and nomenclature of $CD8^+$ T cells.

a Heatmap showing the top10 marker genes of six $CD8^+$ T cell clusters. **b** The violin showing the indicated functional scores of $CD8^+$ T cell subsets estimated by the "AddModuleScore" function in Seurat. Data are presented as mean ±SD.

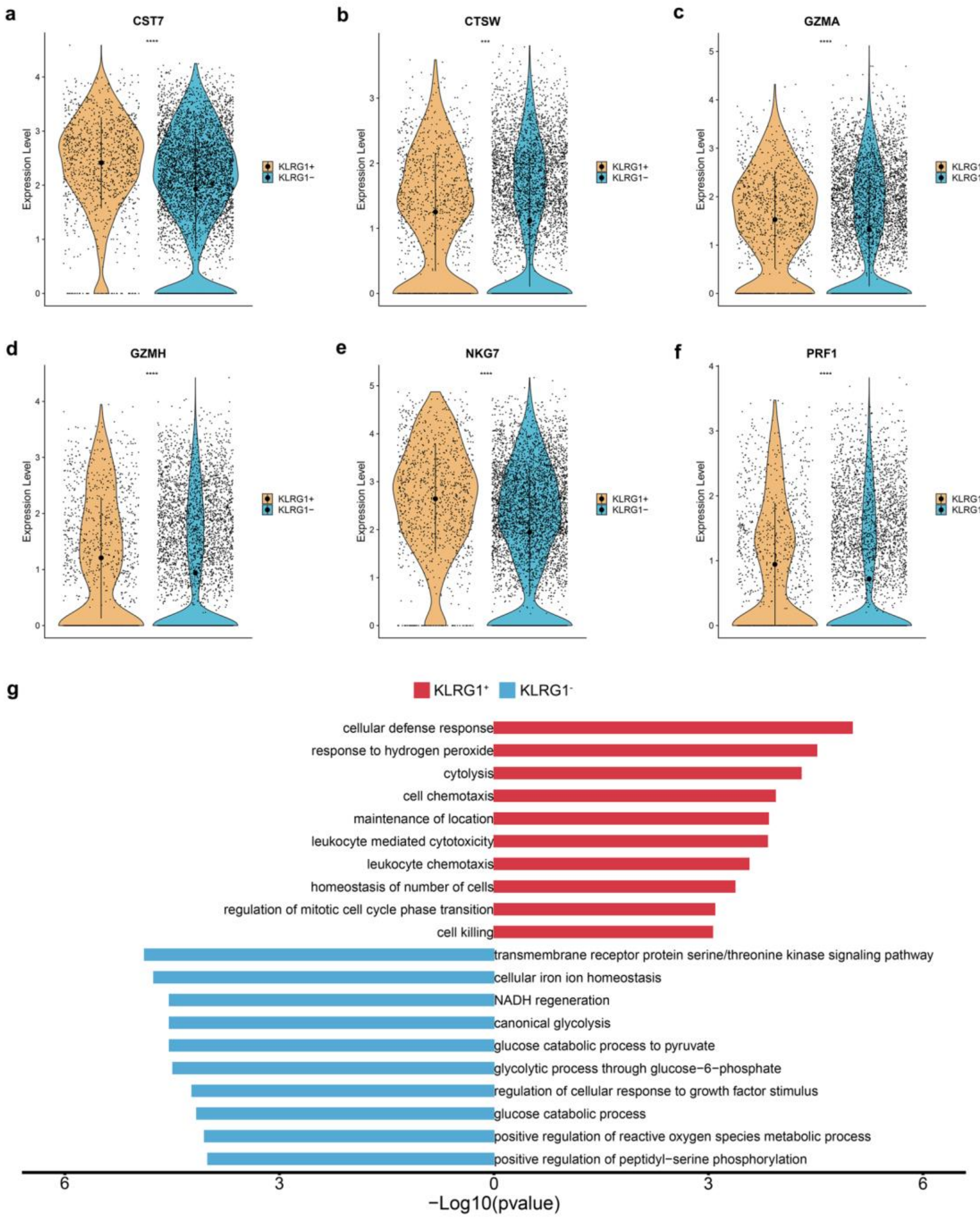

Supplementary Figure 4. Comparison of DEGs and GO functional enrichment analysis between KLRG1$^+$ and KLRG1$^-$ CD8 T cells.

a-f Violin map of cytotoxic genes between KLRG1$^+$ and KLRG1$^-$ CD8 T cells. **g** GO functional enrichment analysis of KLRG1$^+$ and KLRG1$^-$ CD8 T cells.

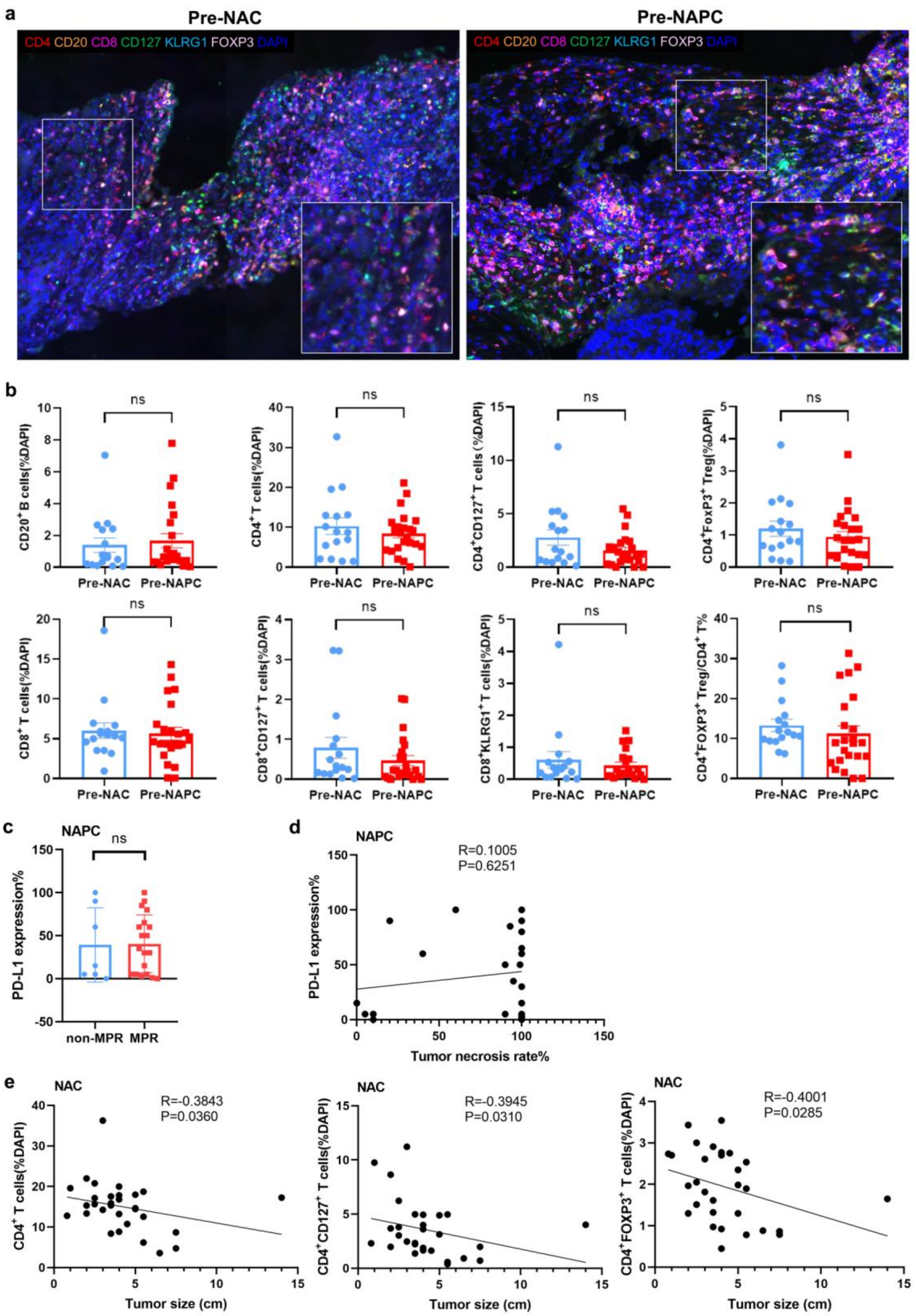

Supplementary Figure 5. Pre-treatment PD-L1 expression and TILs infiltration levels did not correlate with therapy response.

a Representative mIHC staining images of pre-NAC and pre-NAPC samples (Image under 10X). **b** Comparison of $CD20^+$ B cells, $CD4^+$ T cells, $CD4^+CD127^+$ T cells, $CD8^+$ T

cells, $CD8^+CD127^+$ T cells, $CD8^+KLRG1^+$ T cells, $CD4^+FoxP3^+$ Tregs between pre-NAC and pre-NAPC samples. There was no difference in the infiltration of TILs between pre-NAC and pre-NAPC. Pre-NAC: n=16, Pre-NAPC: n=23; P values were determined by Mann-Whitney test, ns: not significant. **c** Comparison of pre-treatment PD-L1 expression in non-MPR and MPR patients of NAPC. **d** Spearman correlation of pre-treatment PD-L1 and tumor necrosis rate. **e** Spearman correlation of tumor size and tumor-infiltrating $CD4^+$ T cells, $CD4^+CD127^+$ T cells and Treg cells in NAC. Data are presented as mean ±SD.

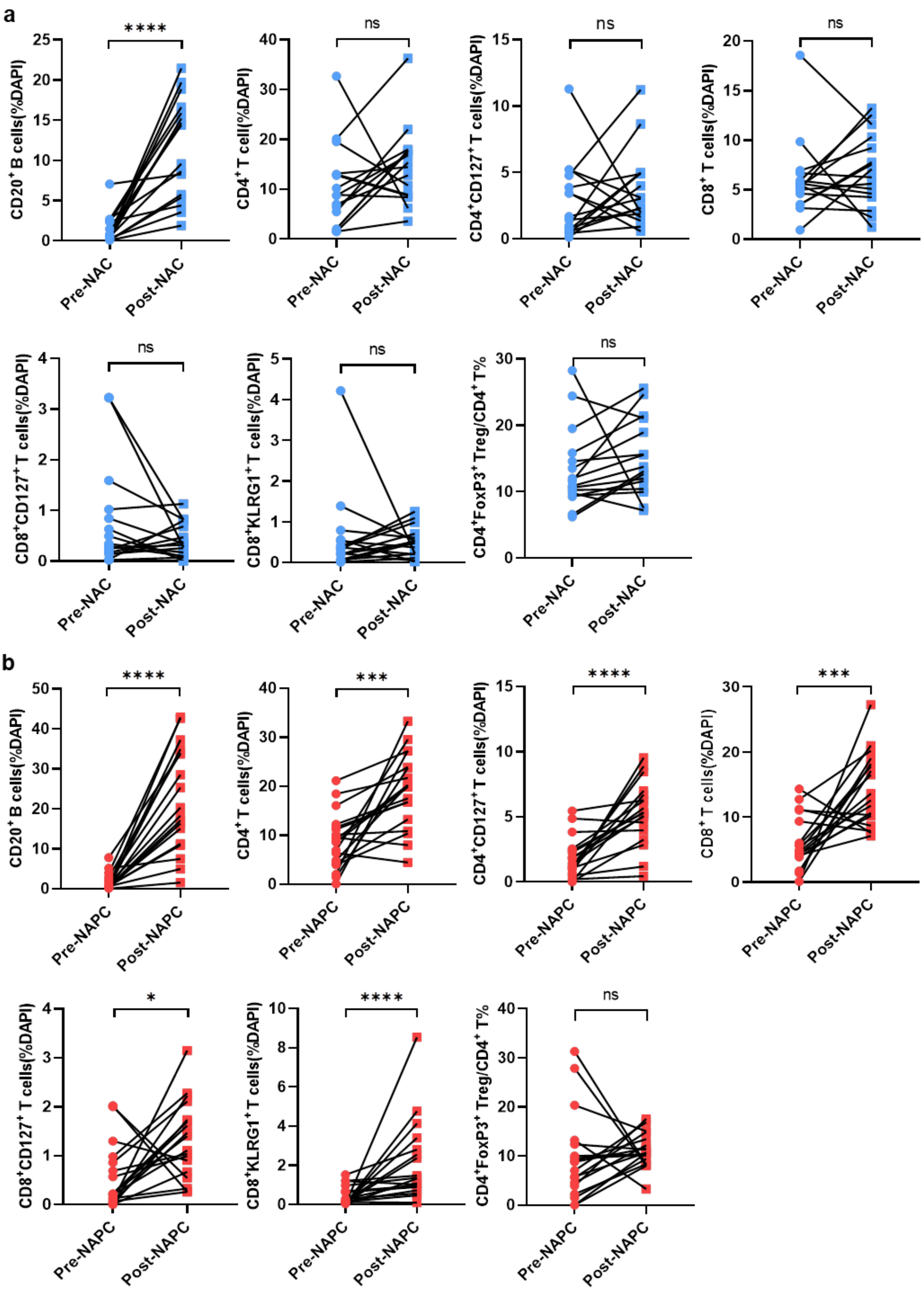

Supplementary Figure 6. Dynamic changes of TILs between self-matched tumors before and after neoadjuvant therapy with NAC or NAPC. NAC：n=16; NAPC: n=18. P values were determined by Wilcoxon matched-pairs signed rank test, * $P < 0.05$; *** $P<0.001$; **** $P < 0.0001$; ns: not significant.

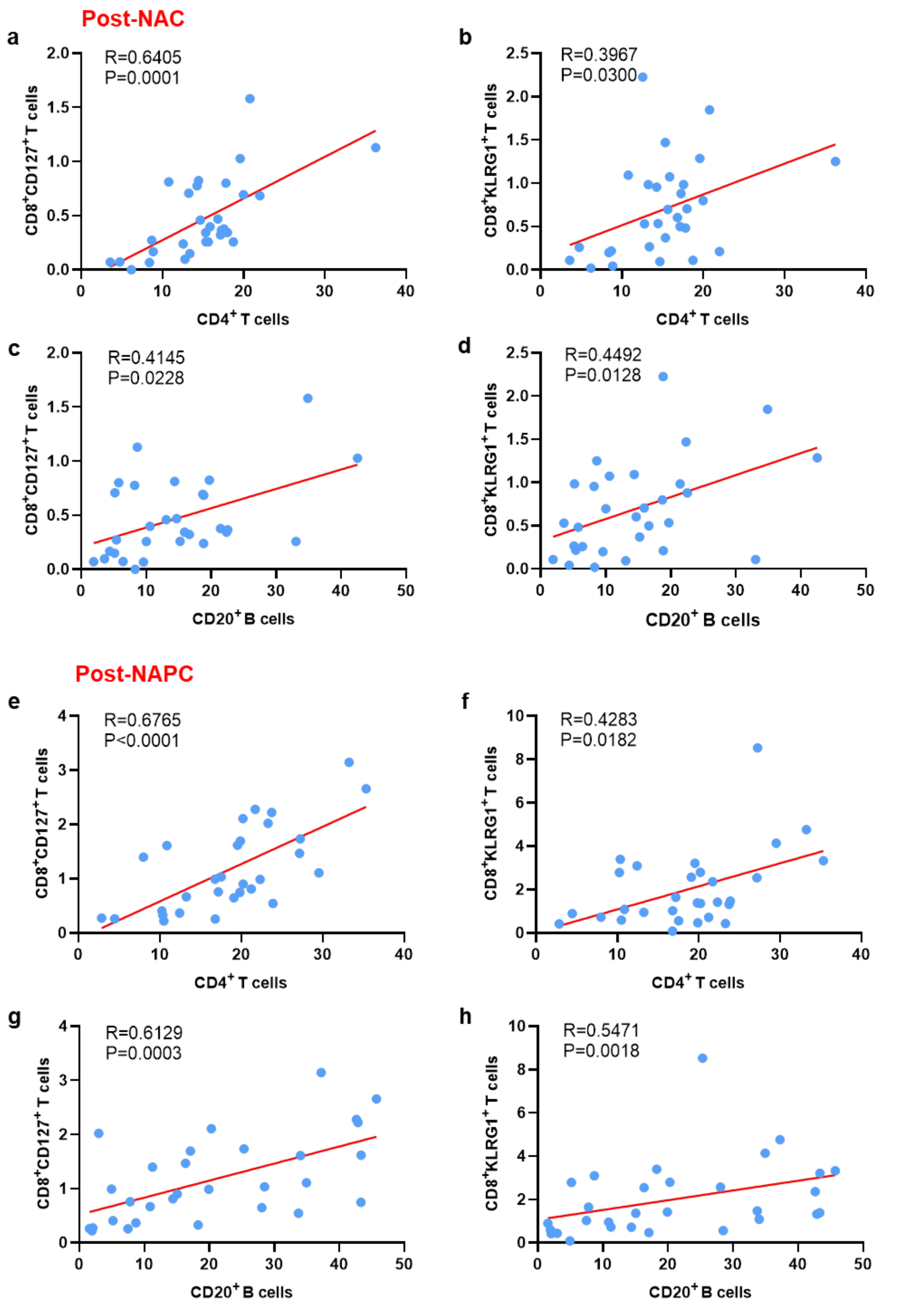

Supplementary Figure 7. Spearman correlation of CD20^{+} B cells and CD4^{+} T cells with CD127^{+} and KLRG1^{+} CD8 T cells in post-NAC and post-NAPC tumor tissues.

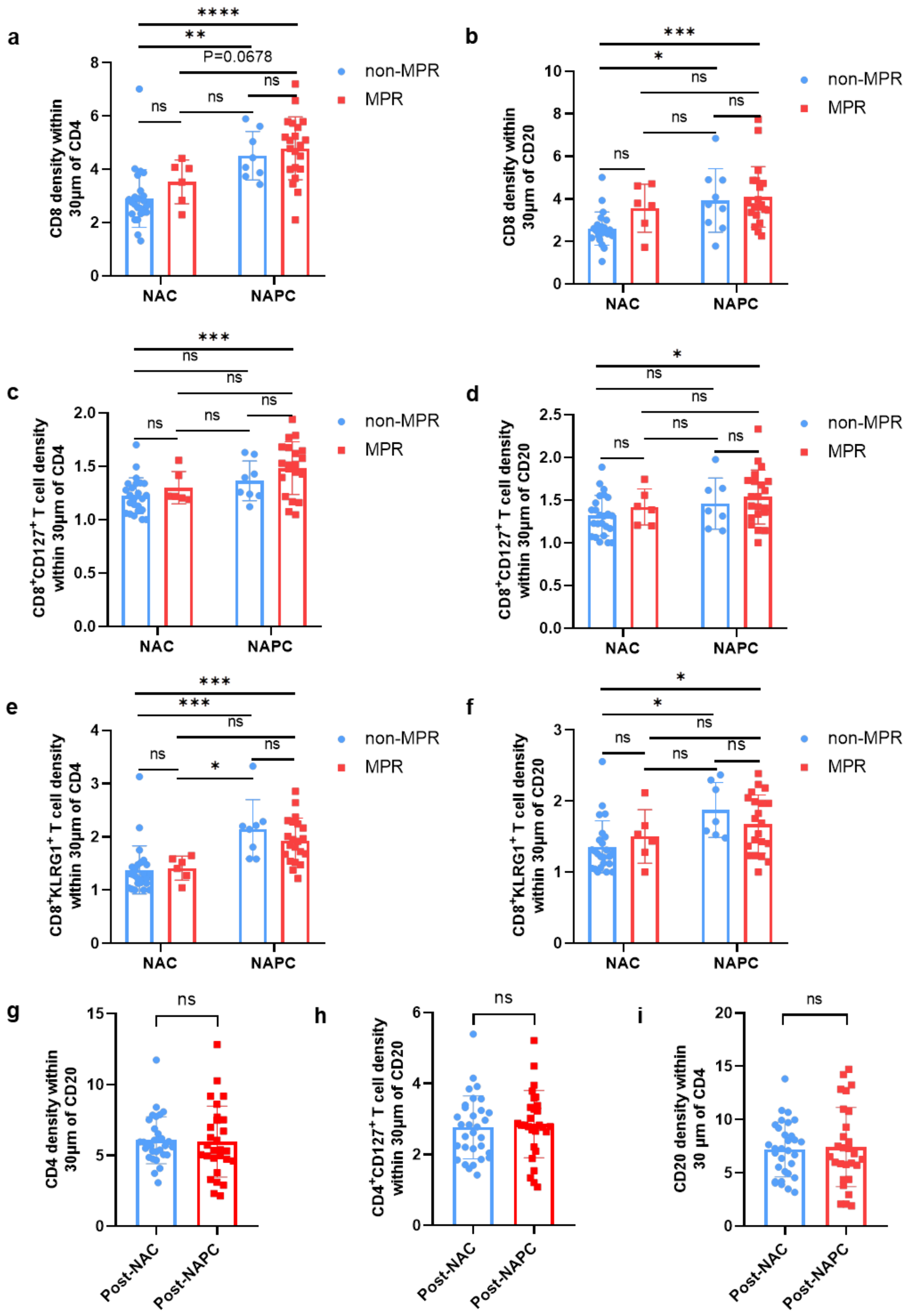

Supplementary Figure 8. Relationship between spatial distribution patterns and pathological response in post-treatment tumor tissues.

a-f Relationship between the spatial distribution of CD8^{+} T, CD8^{+}CD127^{+} and

$CD8^+KLRG1^+$ T cells with $CD4^+$ T and $CD20^+$ B cells and pathological responses. post-NAC-NR n=24, post-NAC-R n=6, post-NAPC-NR n=10, post-NAPC-R n=20; P values were determined by two-way ANOVA with multiple comparisons, * $P < 0.05$; ** $P<0.01$; *** $P<0.001$; **** $P < 0.0001$; ns: not significant. **g-i** The spatial distribution between $CD4^+$ T cells and $CD20^+$ B cells showed no difference in post-NAC and post-NAPC. Post-NAC: n=30, Post-NAPC: n=30. Data are presented as mean ±SD. P values were determined by Mann-Whitney test, ns: not significant.

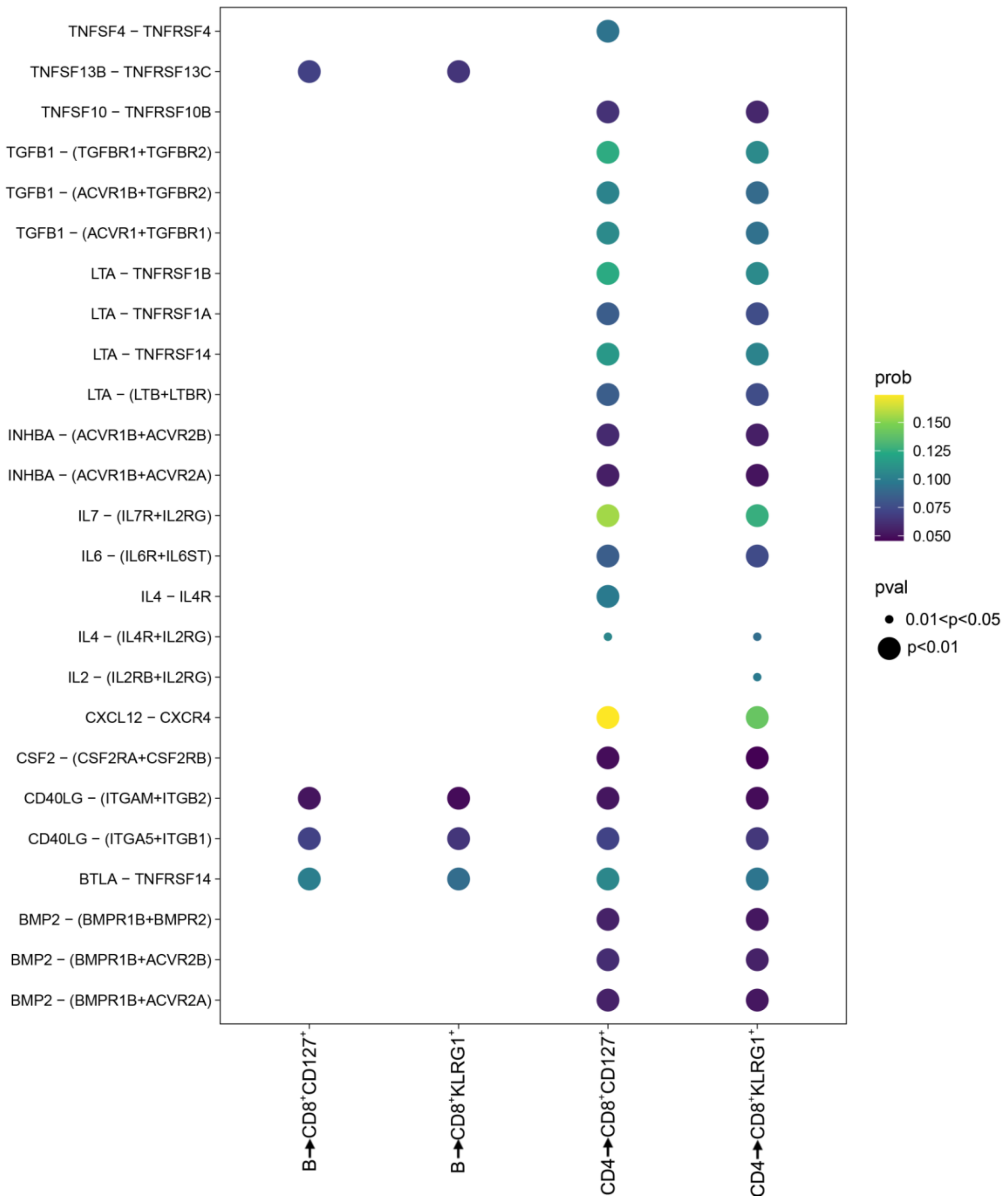

Supplementary Figure 9. Potential ligand-receptor pairs predicted by CellChat for $CD4^+$ T or $CD20^+$ B cells interacting with $CD8^+CD127^+$ and $CD8^+KLRG1^+$ T cells.

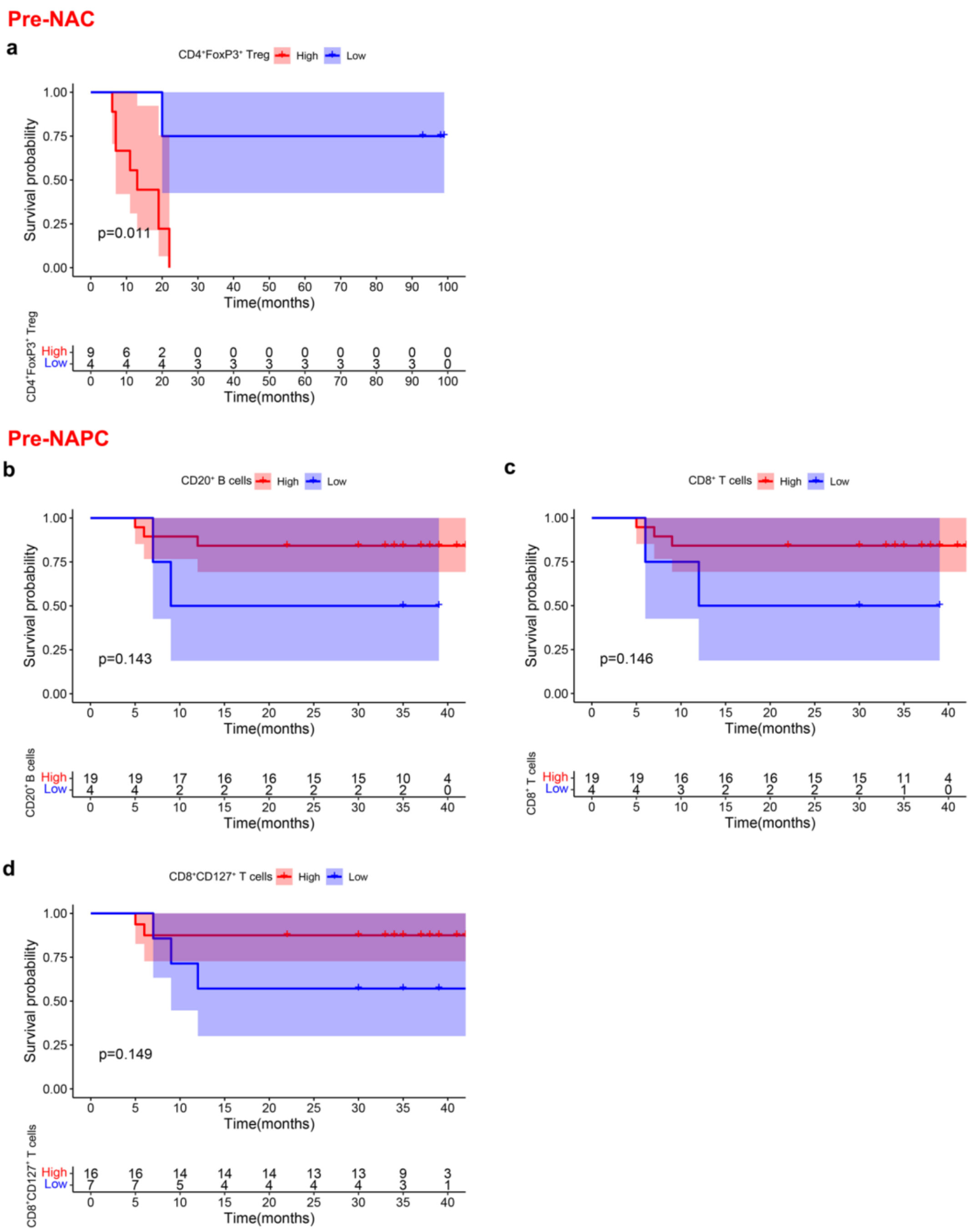

Supplementary Figure 10. Correlation of pre-treatment TILs with clinical outcome.

a Kaplan–Meier estimates of DFS for NAC patients according to the pre-treatment level of $CD4^{+}FoxP3^{+}$ T cells. **b-d** Kaplan–Meier estimates of DFS for NAPC patients according to the pre-treatment level of $CD20^{+}$ B cells, $CD8^{+}$ T cells, and $CD8^{+}CD127^{+}$ T cells. Patients were divided into high and low groups using the package “survminer” of R software.
