# Supplementary material for: PD-1 blockade potentiates neoadjuvant chemotherapy in NSCLC via increasing CD127+ and KLRG1+ CD8 T cells
Source: NPJ Precis Oncol. 2023 May 25;7:48. doi: 10.1038/s41698-023-00384-x (PMC10213055; doi:10.1038/s41698-023-00384-x)
Supplement: Supplementary file 2 — REPORTING SUMMARY [file 41698_2023_384_MOESM2_ESM.pdf]

nature portfolio

Corresponding author(s): Xiubao Ren

Last updated by author(s): Apr 11, 2023

Reporting Summary

Nature Portfolio wishes to improve the reproducibility of the work that we publish. This form provides structure for consistency and transparency in reporting. For further information on Nature Portfolio policies, see our Editorial Policies and the Editorial Policy Checklist.

Statistics

For all statistical analyses, confirm that the following items are present in the figure legend, table legend, main text, or Methods section.

n/a	Confirmed	
☐	☒	The exact sample size (*n*) for each experimental group/condition, given as a discrete number and unit of measurement
☐	☒	A statement on whether measurements were taken from distinct samples or whether the same sample was measured repeatedly
☐	☒	The statistical test(s) used AND whether they are one- or two-sided<br>*Only common tests should be described solely by name; describe more complex techniques in the Methods section.*
☒	☐	A description of all covariates tested
☐	☒	A description of any assumptions or corrections, such as tests of normality and adjustment for multiple comparisons
☐	☒	A full description of the statistical parameters including central tendency (e.g. means) or other basic estimates (e.g. regression coefficient) AND variation (e.g. standard deviation) or associated estimates of uncertainty (e.g. confidence intervals)
☒	☐	For null hypothesis testing, the test statistic (e.g. *F*, *t*, *r*) with confidence intervals, effect sizes, degrees of freedom and *P* value noted<br>*Give P values as exact values whenever suitable.*
☒	☐	For Bayesian analysis, information on the choice of priors and Markov chain Monte Carlo settings
☒	☐	For hierarchical and complex designs, identification of the appropriate level for tests and full reporting of outcomes
☒	☐	Estimates of effect sizes (e.g. Cohen's *d*, Pearson's *r*), indicating how they were calculated

Our web collection on statistics for biologists contains articles on many of the points above.

Software and code

Policy information about availability of computer code

Data collection	10x Genomics, Illumina HiSeq X, Mantra System (PerkinElmer)
Data analysis	Cell Ranger 3.1.0, Seurat 3.2.1, clusterProfiler 3.14.3, ggplot2 3.3.2,dbplyr 1.4.4, CellChat 1.1.3�survival 3.2, survminer 0.4.9, inForm 2.4.4, R software (Version 3.1.0)�Microsoft Excel 2010, SPSS 24.0�GraphPad Prism 8.0.1. Software installation instructions and source code are available at the open source repository.

For manuscripts utilizing custom algorithms or software that are central to the research but not yet described in published literature, software must be made available to editors and reviewers. We strongly encourage code deposition in a community repository (e.g. GitHub). See the Nature Portfolio guidelines for submitting code & software for further information.

Data

Policy information about availability of data

All manuscripts must include a data availability statement. This statement should provide the following information, where applicable:

- Accession codes, unique identifiers, or web links for publicly available datasets
- A description of any restrictions on data availability
- For clinical datasets or third party data, please ensure that the statement adheres to our policy

The datasets generated during and/or analysed during the current study are available from the corresponding author on reasonable request.

Human research participants

Policy information about studies involving human research participants and Sex and Gender in Research.

Reporting on sex and gender	Sex was not considered in our study design, and the sex composition was well balanced in both groups.
Population characteristics	Clinicopathological parameters including age, sex, smoking history, cTNM stage, pathological type, neoadjuvant and adjuvant therapy regimens, type of resection, pathological response, recurrence or not, and follow-up time were obtained (Table 1). Characteristics of patients were generally well balanced between the two groups. Squamous carcinoma was the predominant pathological type in both NAC and NAPC groups, with more than 80% being male and approximately 20% being never smokers.
Recruitment	The inclusion and exclusion criteria for the NAC and NAPC cohorts were consistent, thus the possibility of selection bias is limited. The inclusion and exclusion criteria are shown below:<br>Eligible patients were aged 18 years or older and had histologically treatment-naive and surgically resectable IIA–IIIB NSCLC (American Joint Committee on Cancer 7th edition criteria). All patients had an Eastern Cooperative Oncology Group performance status of 0 or 1, adequate organ function, adequate pulmonary function. Patients were excluded from enrollment if they had a known EGFR mutation or ALK translocation; a history of autoimmune disease, interstitial lung disease or prior cancer; had interstitial lung disease or pneumonitis; receiving ongoing glucocorticoid or immunosuppressant therapy; previously treated with checkpoint inhibitors or other drug that target T-cell co-stimulation or immune checkpoint pathways. Patients with acute or chronic hepatitis virus infection or active tuberculosis were also excluded.
Ethics oversight	The study was approved by the Ethics Committee of Tianjin Cancer Institute & Hospital and all patients enrolled in the study provided written informed consent.

Note that full information on the approval of the study protocol must also be provided in the manuscript.

Field-specific reporting

Please select the one below that is the best fit for your research. If you are not sure, read the appropriate sections before making your selection.

☒ Life sciences ☐ Behavioural & social sciences ☐ Ecological, evolutionary & environmental sciences

For a reference copy of the document with all sections, see nature.com/documents/nr-reporting-summary-flat.pdf

Life sciences study design

All studies must disclose on these points even when the disclosure is negative.

Sample size	We acquired single-cell transcriptomes on a total of 26,861 immune cells from 7 resectable NSCLC patients after neoadjuvant therapy, which was sufficient for data analysis at single-cell level. Sample sizes in mIHC were chosen based on preliminary data demonstrating statistically significant differences between two cohorts.
Data exclusions	Sequencing samples which did not meet the criteria were excluded; Cells with less than 200 or more than 6000 genes detected per cell, or with less than 1000 UMI, or with more than 10% mitochondrial genes were filtered out. Exclusion criterion for mIHC: see above.
Replication	Multiple patients (n≥30) were tested in each group for mIHC.
Randomization	Randomization and covariates were not relevant to our study design.
Blinding	Blinding was not relevant as all processing methods were done through available software with consistent parameters utilized across all treatment groups.

Reporting for specific materials, systems and methods

We require information from authors about some types of materials, experimental systems and methods used in many studies. Here, indicate whether each material, system or method listed is relevant to your study. If you are not sure if a list item applies to your research, read the appropriate section before selecting a response.

Materials & experimental systems

n/a	Involved in the study
☐	☒ Antibodies
☒	☐ Eukaryotic cell lines
☒	☐ Palaeontology and archaeology
☒	☐ Animals and other organisms
☒	☐ Clinical data
☒	☐ Dual use research of concern

Methods

n/a	Involved in the study
☒	☐ ChIP-seq
☒	☐ Flow cytometry
☒	☐ MRI-based neuroimaging

Antibodies

Antibodies used

Pembrolizumab(Keytruda); PD-L1 IHC: anti-human PD-L1 clone 22C3, LOT10145059; For mIHC: Rabbit monoclonal anti-CD4 antibody, Abcam, ab133616, diluted at 1:1000; Rabbit monoclonal anti-CD127 antibody, Abcam, ab259806, diluted at 1:1500; Mouse monoclonal anti-FOXP3 antibody, Abcam, ab20034, diluted at 1:400; Rabbit monoclonal anti-KLRG1 antibody, R&D, MAB70293-SP, diluted at 1:400; Rabbit monoclonal anti-CD8 antibody, Abcam, Ab237710, diluted at 1:800; Rabbit monoclonal anti-CD20 antibody, Abcam, diluted at 1:1000.

Validation

FDA has approved Pembrolizumab(Keytruda) as a first-line treatment in combination with chemotherapy in patients with metastatic non-small cell lung cancer (NSCLC). Anti-human PD-L1 clone 22C3 is recommended for PD-L1 staining to guide clinical treatment. Other antibodies for scientific research only.
